# Composition of Essential Oils from Roots and Aerial Parts of *Carpesium divaricatum*, a Traditional Herbal Medicine and Wild Edible Plant from South-East Asia, Grown in Poland

**DOI:** 10.3390/molecules24234418

**Published:** 2019-12-03

**Authors:** Anna Wajs-Bonikowska, Janusz Malarz, Anna Stojakowska

**Affiliations:** 1 Institute of General Food Chemistry, Faculty of Biotechnology and Food Sciences, Łódź University of Technology, Stefanowskiego street 4/10, 90-924 Łódź, Poland; anna.wajs@p.lodz.pl; 2 Maj Institute of Pharmacology, Polish Academy of Sciences, Department of Phytochemistry, Smętna street 12, 31-343 Kraków, Poland; malarzj@if-pan.krakow.pl

**Keywords:** alpha-pinene, *Carpesium divaricatum*, Inuleae, monoterpenoids, thymol derivatives

## Abstract

*Carpesium divaricatum* Sieb. and Zucc. has long been used both as traditional medicine and seasonal food. The most extensively studied specialized metabolites synthesized by the plant are sesquiterpene lactones of germacrane-type. Low-molecular and volatile terpenoids produced by *C. divaricatum*, however, have never been explored. In this work, compositions of essential oils distilled from roots and shoots of *C. divaricatum* plants, cultivated either in the open field or in the glasshouse have been studied by GC-MS-FID supported by NMR spectroscopy. The analyses led to the identification of 145 compounds in all, 112 of which were localized in aerial parts and 80 in roots of the plants grown in the open field. Moreover, remarkable differences in composition of oils produced by aerial and underground parts of *C. divaricatum* have been observed. The major volatiles found in the shoots were: α-pinene (40%), nerol (4%) and neryl-isobutyrate (3%), whereas predominant components of the root oil were 10-isobutyryloxy-8,9-epoxythymyl-isobutyrate (29%), thymyl-isobutyrate (6%) and 9-isobutyryloxythymyl-isobutyrate (6%). In the analyzed oils, seventeen thymol derivatives were identified. Among them eight compounds were specific for roots. Roots of the plants cultivated in the glasshouse were, in general, a poor source of essential oil in comparison with those of the plants grown in the open field. Chemophenetic relationships with other taxa of the Inuleae-Inulineae were also briefly discussed.

## 1. Introduction

Plant genera of the subtribe Inuleae-Inulinae (family Asteraceae), e.g., *Inula*, *Pulicaria*, *Telekia*, *Dittrichia*, *Blumea* and *Chiliadenus*, are known to produce essential oils containing biologically active mono- and sesquiterpenoids [1,2,3]. Essential oils from *Carpesium* spp. are less studied. To our knowledge, only two communications on essential oil from the herb of *C. abrotanoides* L., the species included in the Chinese pharmacopoeia, have been published to date [4,5]. *C. divaricatum* Sieb. and Zucc. is a medicinal and food plant rich in terpenoid metabolites [6,7,8,9]. Recently, hydroxycinnamates and biologically active oxylipin from aerial parts of the plant have been also described [10]. According to the recent taxonomic studies [11,12], *Telekia speciosa* (Schreb.) Baumg. as well as some species of the genus *Inula*, known as essential oil bearing plants, are closely related to *C. divaricatum*. However, the content and composition of essential oils from *C. divaricatum* has remained unknown until now. The aim of the present study was to investigate the volatile compounds from roots and aerial parts of *C. divaricatum* and to compare the newly generated data with those reported previously for the related species.

## 2. Results

**Figure 1:**
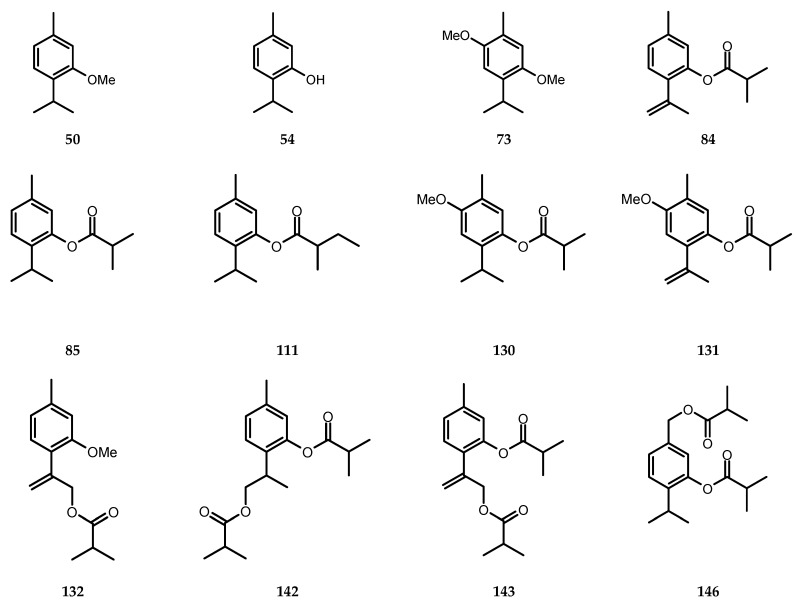

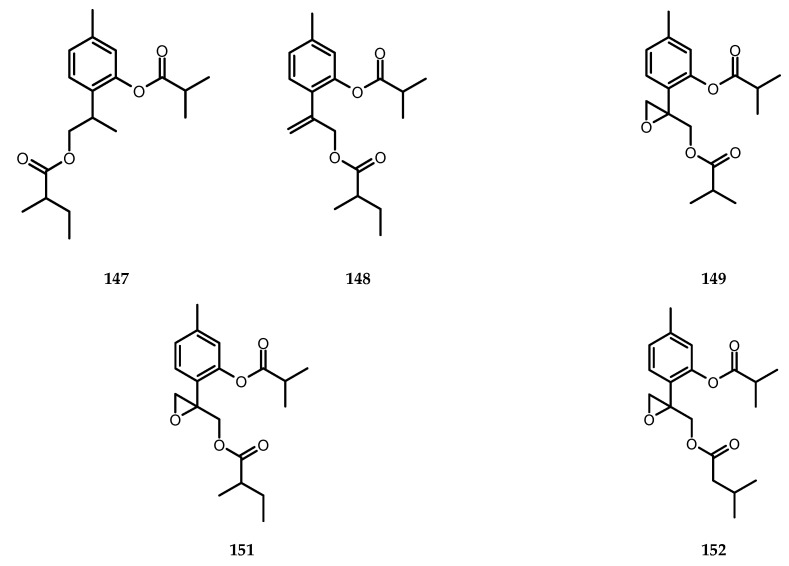
Structures of thymol derivatives identified in essential oils from roots of *Carpesium divaricatum*. **50**: thymol-methyl-ether; **54**: thymol; **73**: 6-methoxythymol-methyl-ether; **84**: 8,9-didehydrothymyl-isobutyrate; **85**: thymyl-isobutyrate; **111**: thymyl-2-methylbutyrate; **130**: 6-methoxythymyl-isobutyrate; **131**: 6-methoxy-8,9-didehydrothymyl-isobutyrate; **132**: 10-isobutyryloxy-8,9-didehydrothymol-methyl-ether; **142**: 9-isobutyryloxythymyl-isobutyrate; **143**: 10-isobutyryloxy-8,9-didehydrothymyl-isobutyrate; **146**: 7-Isobutyryloxythymyl-isobutyrate; **147**: 9-(2-methylbutyryloxy)-thymyl-isobutyrate; **148**: 10-(2-methylbutyryloxy)-8,9-didehydrothymyl-isobutyrate; **149**: 10-isobutyryloxy-8,9-epoxythymyl-isobutyrate; **151**: 10-(2-methylbutyryloxy)-8,9-epoxythymyl-isobutyrate; **152**: 10-isovaleryloxy-8,9-epoxythymyl-isobutyrate.

**Figure 2:**
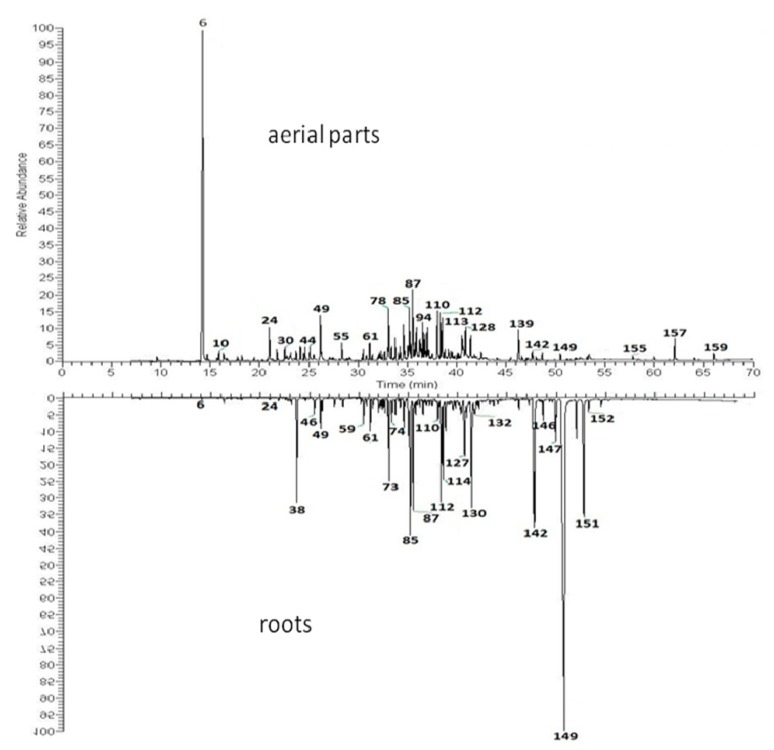
Gas chromatograms of essential oils from aerial parts and roots of *Carpesium divaricatum*. The numbering of the compounds corresponds to that in Table 1.

Unlike in its natural habitat, in our climate *C. divaricatum* is an annual plant. Moreover, due to late flowering, the plants grown in the open field failed to produce seeds. Fertile seeds were obtained only from the plants cultivated in a glasshouse. Yields of essential oils produced by the aerial parts of the plants were low (<0.02%, see Table 1) and except for the variations in percentages of individual compounds, oils distilled from shoots of the field grown plants and from the aerial parts of plants cultivated in a glasshouse demonstrated some minor qualitative differences in their composition (112 versus 89 identified constituents). The major compounds found in the essential oil from shoots of *C. divaricatum* were: α-pinene (c. 40% of oil), nerol (2.1%–3.7%) and neryl isobutyrate (3.2%–3.9%). Identified thymol derivatives (compounds: **54**, **73**, **84**, **85**, **111**, **130**, **142**, **148** and **149**, see Figure 1) constituted c. 6% of the oil. Roots of the plant turned out to be much better source of volatile terpenoids (yield of essential oil—0.15%). In contrast to the aerial parts (Figure 2), they contained only low amount of α-pinene (up to 1.8% of the essential oil). Thymol derivatives (17 identified structures, see Figure 1) accounted for over 60% and 44% of the essential oil from roots of the garden grown plants and plants cultivated in the glasshouse, respectively. 10-Isobutyryloxy-8,9-epoxythymyl isobutyrate was the major constituent of the analyzed root oils (18.1%–29.2%).

The essential oils from *C. divaricatum* contained some volatiles, which were difficult to identify based on GC-MS only. Flash chromatography (FC), monitored by thin-layer chromatography (TLC), was used to obtain fractions of oils rich in components of interest (purity 19%–63%, by GC-FID). The fractions were subsequently subjected to NMR analysis and the experimental chemical shifts of the chosen volatiles were compared to the literature data (see Supplementary Material).

Structures of 11 components remained unresolved, due to the small available amounts of the compounds, insufficient to perform full spectral analysis. MS spectra and retention indices of the compounds are shown in Supplementary Material.

## 3. Discussion

Though the essential oil content in aerial parts of *C. divaricatum* was very low, the occurrence of α-pinene (40% of the oil) is worth to note. The compound demonstrated anxiolytic and moderate anti-inflammatory effect in mice [13,14]. Essential oils obtained from plants of different provenience can markedly vary in their composition. Aerial parts of *Pulicaria gnaphalodes* (Vent.) Boiss., collected in four different locations, contained extremely different quantities of α-pinene (0.0–34.1% of the essential oil) [15]. Thus, some data on the composition of essential oils from *C. divaricatum* plants grown in their natural habitat would be of interest, to establish whether or not the high α-pinene content is typical of *C. divaricatum* aerial parts. Not much is known from the literature on essential oils from plants of the genus *Carpesium*. To date, only two studies on volatiles from the whole herb of *C. abrotanoides* have been published [4,5]. However, the authors managed to identify 14–44 components of the oils and neither α-pinene nor thymol derivatives have been detected. The major constituents were β-bisabolene (7.3–24.7%), caryophyllene-oxide (c. 13%) and eudesma-5,11(13)-dien-8,12-olide (c. 22%). Volatile constituents from other species of the Inuleae-Inulinae subtribe are better investigated. Thymol and its derivatives seem to be widespread within the plants of the subtribe, except for *Blumea* spp. [16,17,18]. The genus *Pulicaria* comprises species with essential oils rich in thymol and its methyl ether, like *Pulicaria vulgaris* Gaertn. [19] and *Pulicaria sicula* (L.) Moris [15] together with some species devoid of thymol derivatives [20]. The content of thymol derivatives in essential oil from aerial parts of *C. divaricatum* (6.4%) is similar to those detected in oils from aerial parts of other species of the subtribe, e.g., *Schizogyne sericea* (L.F.) DC. [21,22], *Telekia speciosa* (Schreb.) Baumg. [23,24] and *Limbarda crithmoides* (L.) Dumort. (formerly *Inula crithmoides* L.) [25]. Structural diversity of the compounds was also similar, with numerous thymol esters.

Essential oils from roots of the Inuleae-Inulinae plants have rarely been studied. Literature data on a few species are available, including *Dittrichia viscosa* (L.) Greuter (formerly *Inula viscosa* (L.) Aiton) [26], *Inula racemosa* Hook. f. [27,28], *Inula helenium* L. [1], *Pulicaria mauritanica* Coss. [29] and *T. speciosa* [24,30]. The common feature of essential oils from *I. helenium*, *I. racemosa* and *T. speciosa* is a very high content of eudesmane-type sesquiterpene lactones (up to 82%). Such composition of essential oils seems to be correlated with a presence of resin canals in roots of the plants. Thymol derivatives were not described as constituents of essential oil from roots of *I. racemosa*. The compounds, however, were found in the oils from the remaining species. Juvenile roots of *I. helenium* and *I. viscosa* contained higher amounts of the monoterpenoids than the old ones [26,31]. Two derivatives of thymol methyl ether constituted nearly 80% of the volatile fraction from *I. viscosa* roots [26]. Thymol, thymol methyl ether and eight thymyl ester derivatives accounted for c. 5.5% of the essential oil from roots of *T. speciosa*. *P. mauritanica* root oil contained c. 16% of the structurally related compounds. Though there are no any data on essential oils from roots of *Carpesium* spp., some thymol derivatives were described as constituents of methanol extract from aerial parts of *C. divaricatum* [32]. All of the compounds were found in essential oils from the plants analyzed in this study. Volatile fraction from roots of *C. divaricatum* is exceptional, in respect of both thymol derivatives content (over 60%) and their structural diversity (17 compounds; for MS spectra see Supplementary Material). 10-Isobutyryloxy-8,9-epoxy-thymyl isobutyrate, major constituent of the analyzed essential oil, demonstrated moderate activity against *Staphylococcus aureus* and *Candida albicans* [33].

## 4. Materials and Methods 

### 4.1. General Experimental Procedures

GC-MS-FID analyses of essential oils and their fractions were performed on a Trace GC Ultra Gas Chromatograph coupled with DSQII mass spectrometer (Thermo Electron, Waltham, MA, USA). Simultaneous GC-FID and GC-MS analysis were performed using a MS-FID splitter (SGE Analytical Science, Ringwood, VIC, Australia). Mass range was 33–550 amu, ion source-heating: 200 °C; ionization energy: 70 eV. One microliter of essential oil solution (80% *v*/*v*) diluted in pentane:diethyl ether was injected in split mode at split ratios (50:1). Operating conditions: capillary column Rtx-1 MS (60 m × 0.25 mm i.d., film thickness 0.25 μm), and temperature program: 50 °C (3 min)—300 °C (30 min) at 4 °C/min. Injector and detector temperatures were 280 °C and 300 °C, respectively. Carrier gas was helium (constant pressure: 300 kPa). The relative composition of each essential oil sample was calculated from GC peak areas according to total peak normalization—the most popular method used in the essential oil analysis. ^1^H-NMR (250 MHz) and ^13^C-NMR (62.90 MHz) spectra for components of essential oils were recorded with a Bruker DPX 250 Avance spectrometer in CDCl_3_, with TMS as an internal standard.

### 4.2. Plant Material

Seeds of *Carpesium divaricatum* Sieb. and Zucc, provided by the Research Center for Medicinal Plant Resources, National Institute of Biomedical Innovation, Tsukuba (Japan), were sown in the end of March 2015, into multipots with garden soil. In the stage of 4–5 mature leaves, the plants were transferred to plastic pots with a substrate composed of garden soil, peat and sand (2:1:1, *v*/*v*). Plants were grown in a glasshouse of the Garden of Medicinal Plants, Maj Institute of Pharmacology PAS in Krakow, under controlled conditions (temperatures by day 18–38 °C; by night 12–18 °C), without any chemical treatment. In the third week of May, the plants were divided into two groups. First group was left in the glasshouse for further growth and the second one was transplanted into the open field. Data on cultivation conditions (type of soil, average annual temperature, annual rainfall and agrotechnical procedures applied) are available elsewhere [34]. Aerial parts and roots of the plants were collected in the beginning of flowering period (August/September) and dried under shade at room temperature. Voucher specimen (3/15) was deposited in the collection kept at the Garden of Medicinal Plants, Institute of Pharmacology, Kraków, Poland. The dry plant material was stored no longer than five months.

### 4.3. Isolation of Essential Oil

Essential oils from aerial (dried leaf, branches, flowers) and underground parts (dried roots) of *C. divaricatum* were obtained by hydrodistillation using a Clevenger-type apparatus. Each hydrodistillation was conducted for 4 h using 100–300 g of plant material. The yellowish essential oils were dried over anhydrous magnesium sulphate, and stored at 4 °C in the dark, until tested and analyzed.

### 4.4. Isolation and NMR Analysis of Volatile Components

To isolate the volatiles of interest, the essential oils from aerial parts (i.e., dried leaves with petioles, stems and flowers, 504 mg) and from roots (dried plant material, 973 mg) of the plants grown in the open field were separately flash-chromatographed (FC) on a glass column (500 × 30 mm) filled with silica gel 60 (0.040–0.063 mm, Merck, EM Science, NJ USA), starting the elution with *n*-hexane and gradually increasing the polarity by addition of diethyl ether. The elution was accelerated by means of pressurized nitrogen (flow rate 100 mL/min). The separation was monitored by TLC and GC-MS. Twenty fractions (1a–20a) of essential oil distilled from the aerial parts of the plant and twenty fractions (1b–20b) of root essential oil were obtained and analyzed by GC-MS-FID. Structures of 11 volatiles from the following fractions were confirmed using NMR spectroscopy (^1^H and/or ^13^C; see Supplementary Material): 1a: (42 mg) neryl-isobutyrate (26%); 13a: (22mg) (*E*)-nerolidol (25%); 15a: (22 mg) τ-cadinol (21%); 17a: (58 mg) nerol (25%); 18a: (48 mg) α-cadinol (23%); 3b: (17 mg) thymol-methyl-ether (33%); 7b: (32 mg) thymyl isobutyrate (57%); 8b: (51 mg) 6-methoxythymyl-isobutyrate (62%); 13b: (13 mg) caryophyllene-oxide (52%); 14b: (36%) 10-isobutyryloxy-8,9-didehydrothymyl-isobutyrate (46%); 15b: (65 mg) 9-isobutyryloxythymyl-isobutyrate (55%); 17b: (53 mg) 10-isobutyryloxy-8,9-epoxythymyl-isobutyrate (63%); 18b: (25 mg) nerol (43%).

### 4.5. Identification of Essential Oil Constituents

Constituents of the essential oils were identified based on their MS spectra and their comparison with those from mass spectra libraries: NIST 2012, Wiley Registry of Mass Spectral Data 8th edition and MassFinder 4.1, along with the relative retention indices (RI) on DB-1 column (available from MassFinder 4.1) and on Rtx-1MS column found in the literature [35]. Isolated compounds were also identified by the comparison of their ^1^H-NMR and ^13^C-NMR spectral data with those of the compounds isolated previously in our laboratory or those from the literature.

## 5. Conclusions

This was the first study on composition of essential oils from *C. divaricatum*. Aerial parts of *C. divaricatum* occurred to be a poor source of volatiles. Essential oil from roots of the plant was rich in thymyl ester derivatives of various structures. As some of the compounds, according to the literature [36], demonstrated moderate antibacterial, antifungal and anti-inflammatory activities, the essential oil from roots of *C. divaricatum* as well as its components are worth further studies.

## Figures and Tables

**Table 1:** Chemical composition of essential oils from aerial parts and roots of *Carpesium divaricatum*.

No	Compound	Amount (%)				RI ^c^ exp.	RI ^d^ lit.	Identification Method
		Aerial Parts		Roots				
		OF ^a^	G ^b^	OF ^a^	G ^b^			
1	hexanal	-	-	0.1	-	771	771	RI ^e^, MS ^f^
2	(*E*)-hex-2-enal	0.2	-	-	-	825	832	RI, MS
3	hexan-1-ol	0.1	-	tr.	-	852	837	RI, MS
4	tricyclene	tr.	-	-	-	917	927	RI, MS
5	α-thujene	0.1	-	-	-	922	932	RI, MS
6	**α-pinene**	**40.2**	**21.8**	0.1	**1.8**	930	936	RI, MS
7	camphene	0.3	-	-	-	940	950	RI, MS
8	sabinene	tr.	-	-	-	944	973	RI, MS
9	6-methylhept-5-en-2-one	0.2	0.2	-	-	962	978	RI, MS
10	**β-pinene**	**0.5**	0.3	-	tr.	966	978	RI, MS
11	2-pentylfuran	0.4	0.3	0.2	-	976	981	RI, MS
12	*trans*-2-(pent-2-enyl)furan	0.1	0.1	-	-	984	984	RI, MS
13	α-phellandrene	-	-	tr.	-	991	1002	RI, MS
14	δ-car-3-ene	tr.	-	-	-	1005	1010	RI, MS
15	m-cymene	0.2	tr.	tr.	-	1006	1013	RI, MS
16	p-cymene	-	-	tr.	-	1007	1015	RI, MS
17	β-phellandrene	-	-	tr.	-	1014	1023	RI, MS
18	limonene	0.2	tr.	-	0.1	1018	1025	RI, MS
19	γ-terpinene	0.1	0.2	-	-	1047	1051	RI, MS
20	*trans*-linalool oxide (furanoid)	tr.	-	tr.	-	1055	1058	RI, MS
21	camphen-6-ol	tr.	-	-	-	1066	1082	RI, MS
22	terpinolene	tr.	tr.	-	-	1077	1082	RI, MS
23	n-nonanal	-	0.1	-	-	1080	1076	RI, MS
24	**linalool**	**2.1**	**3.4**	0.1	tr.	1083	1086	RI, MS
25	145/89/143/115 M?	0.1	-	-	-	1091	-	RI, MS
26	limona ketone	-	-	tr.	-	1100	1105	RI, MS
27	**α-campholenal**	**0.6**	0.1	-	-	1101	1105	RI, MS
28	*cis*-p-menth-2-en-1-ol	0.1	tr.	0.1	0.1	1104	1108	RI, MS
29	*trans*-p-menth-2-en-1-ol	-	-	0.1	0.1	1119	1116	RI, MS
30	***trans*-pinocarveol**	**0.7**	0.2	-	-	1120	1126	RI, MS
31	*cis*-verbenol	-	tr.	-	-	1121	1132	RI, MS
32	*trans*-verbenol	0.3	0.2	-	-	1124	1134	RI, MS
33	2-hydroxy-3-methyl- benzaldehyde	-	-	0.1	0.2	1126	1135	RI, MS
34	(*E*)-non-2-enal	0.1	tr.	0.1	0.2	1133	1136	RI, MS
35	**nerol oxide**	-	-	0.2	**1.0**	1134	1137	RI, MS
36	**pinocarvone**	**0.5**	0.1	-	-	1135	1137	RI, MS
37	*p*-mentha-1,5-dien-8-ol	0.1	0.2	-	-	1143	1138	RI, MS
38	**geijeren**	0.4	tr.	**4.2**	**3.1**	1148	1139	RI, MS
39	**terpinen-4-ol**	**0.7**	**0.9**	-	-	1159	1164	RI, MS
40	myrtenal	-	tr.	-	-	1165	1172	RI, MS
41	**α-terpineol**	**0.8**	**0.7**	0.1	0.1	1171	1176	RI, MS
42	*cis*-piperitol	-	-	tr.	tr.	1176	1181	RI, MS
43	myrtenol	0.1	-	-	-	1177	1178	RI, MS
44	**n-decanal**	**0.6**	**1.2**	-	-	1182	1180	RI, MS
45	*trans*-piperitol	-	-	tr.	tr.	1186	1193	RI, MS
46	**2-ethenyl-3-methyloanisol**	0.2	-	**0.7**	**0.9**	1190	1196	RI, MS
47	β-cyclocitral	0.2	0.3	-	-	1193	1195	RI, MS
48	*trans*-carveol	0.1	-	-	-	1195	1200	RI, MS
49	**nerol**	**3.7**	**2.1**	**1.4**	**1.2**	1210	1210	^1^H, RI, MS
50	thymol methyl ether	-	-	0.4	0.1	1211	1215	^1^H, RI, MS
51	geraniol	0.2	0.4	-	-	1233	1235	RI, MS
52	α-jonene	0.1	-	-	-	1241	1258	RI, MS
53	cuminol	tr.	-	0.3	0.3	1245	1266	RI, MS
54	thymol	0.1	-	0.1	0.1	1258	1267	RI, MS
55	**carvacrol**	**0.9**	-	0.3	0.3	1264	1278	RI, MS
56	dihydroedulan II	0.1	0.1	-	-	1278	1290	RI, MS
57	(*E,E*)-deca-2,4-dienal	0.1	-	-	0.1	1286	1291	RI, MS
58	4,6-dimethyl-2,3-2H- benzofuran-2-one	-	-	0.2	0.2	1317	-	RI, MS
59	**7αH-silphiperfol-5-ene**	**0.5**	0.1	**0.7**	**2.5**	1323	1329	RI, MS
60	presilphiperfol-7-ene	0.2	-	0.2	0.3	1332	1342	RI, MS
61	**7βH-silphiperfol-5-ene**	**0.9**	0.1	**1.1**	**3.4**	1342	1352	RI, MS
62	α-cubebene	tr.	0.1	-	-	1344	1355	RI, MS
63	α-longipinene	0.2	tr.	0.3	0.4	1348	1360	RI, MS
64	(*E*)-tridec-6-en-4-yn	0.2	0.1	-	-	1363	-	RI, MS
65	**viburtinal**	-	-	**0.5**	**1.2**	1367	-	RI, MS
66	longicyclene	0.4	-	0.3	0.4	1371	1372	RI, MS
67	cyclosativene	0.4	-	-	-	1372	1378	RI, MS
68	**α-copaene**	-	**0.5**	-	-	1375	1379	RI, MS
69	silphiperfol-6-ene	-	-	0.3	0.4	1376	1379	RI, MS
70	**modephene**	0.2	-	0.3	**1.0**	1377	1383	RI, MS
71	**α-isocomene**	0.4	0.3	0.4	**1.3**	1383	1389	RI, MS
72	**137/121/95/136 M204**	**0.6**	0.1	**0.5**	**0.8**	1391	-	RI, MS
73	**6-methoxythymol methyl-ether**	**2.1**	**0.5**	**2.7**	**1.4**	1394	1398	RI, MS
74	**β-isocomene**	**0.6**	0.2	**0.7**	**2.0**	1402	1411	RI, MS
75	α-cedrene	0.1	-	tr.	tr.	1409	1418	RI, MS
76	α-gurjunene	-	tr.	-	-	1410	1418	RI, MS
77	**α-santalene**	**1.0**	**0.5**	**0.6**	**0.8**	1413	1422	RI, MS
78	*trans*-geranylacetone	0.3	0.3	-	-	1423	1430	RI, MS
79	***trans*-α-bergamotene**	**0.6**	0.1	0.3	0.2	1428	1434	RI, MS
80	***epi*-β-santalene**	**1.5**	0.4	**0.9**	**0.8**	1438	1446	RI, MS
81	**α-himachalene**	**0.5**	0.1	-	-	1441	1450	RI, MS
82	aromadendrene	0.2	-	0.3	0.4	1442	1449	RI, MS
83	α-humulene	0.1	-	tr.	tr.	1446	1455	RI, MS
84	**8,9-didehydrothymyl-** **isobutyrate**	**0.9**	0.2	**1.6**	**0.8**	1461	1458	RI, MS
85	**thymyl-isobutyrate**	**2.0**	**1.0**	**6.3**	**3.5**	1467	1462	^1^H, RI, MS
86	**β-jonone**	**0.9**	**1.5**	-	-	1468	1468	RI, MS
87	**neryl isobutyrate**	**3.2**	**3.9**	**4.1**	**3.9**	1475	1468	^1^H, RI, MS
88	γ-himachalene	0.3	0.1	-	-	1480	1479	RI, MS
89	**123/93/94/121 M204**	0.8	0.1	**0.5**	**0.7**	1484	-	RI, MS
90	**(3*E*,6*Z*)-α-farnesene**	**1.8**	**5.5**	-	-	1487	1475	RI, MS
91	**α-terpinyl isovalerate**	-	-	0.2	**0.7**	1489	1488	RI, MS
92	γ-muurolene	tr.	0.2	-	-	1493	1494	RI, MS
93	elixene (4-isopropylidene-1-vinyl-*o*- menth-8-ene)	-	-	0.2	0.2	1498	1493	RI, MS
94	**ledene**	**1.5**	**2.2**	tr.	0.2	1499	1491	RI, MS
95	**α-muurolene**	-	**1.1**	-	-	1500	1496	RI, MS
96	**(*E*,*E*)-α-farnesene**	**0.7**	**1.1**	-	-	1502	1498	RI, MS
97	**β-bisabolene**	**1.6**	**0.6**	0.5	0.4	1507	1503	RI, MS
98	**γ-cadinene**	**1.1**	**3.2**	-	-	1511	1507	RI, MS
99	cameronan-7α-ol	-	-	tr.	0.1	1513	1513	RI, MS
100	α-photosantalol	-	-	0.1	0.2	1514	1514	RI, MS
101	**isolongifolan-8-ol**	-	-	0.1	**0.5**	1517	1515	RI, MS
102	*cis*/*trans*-calamenene	0.2	0.4	0.2	0.3	1526	1517	RI, MS
103	**δ-cadinene**	**1.4**	**5.3**	-	-	1520	1520	RI, MS
104	β-cadinene	0.1	0.3	-	-	1523	1526	RI, MS
105	9-methoxycalamenene	0.1	-	-	-	1524	-	RI, MS
106	147/162/121/177 M206	-	-	tr.	0.1	1531	-	RI, MS
107	121/163/93/134 M218	-	-	0.2	0.1	1534	-	RI, MS
108	**α-cadinene**	0.3	**0.5**	-	-	1535	1534	RI, MS
109	**(*E*)-α-bisabolene**	0.1	0.2	0.1	**0.7**	1536	1530	RI, MS
110	**(*E*)-nerolidol**	**2.2**	**8.6**	**0.6**	**0.6**	1543	1553	^1^H, RI, MS
111	thymyl-2-methylbutyrate	0.1	-	0.2	0.2	1546	-	RI, MS
112	**neryl-α-methylbutyrate**	**1.7**	**1.6**	**3.6**	**3.4**	1551	1565	RI, MS
113	**neryl isovalerate**	**1.6**	**1.3**	**2.3**	**1.8**	1557	1579	RI, MS
114	**caryophyllene oxide**	0.4	0.4	**1.1**	**2.1**	1565	1578	^1^H, RI, MS
115	**viridiflorol**	**0.5**	**1.4**	-	-	1577	1592	RI, MS
116	isoaromadendreneepoxide	0.3	0.1	-	-	1584	1590	RI, MS
117	**ledol**	0.3	**0.5**	-	-	1588	1600	RI, MS
118	**humulene II epoxide**	-	-	0.4	**1.1**	1595	1602	RI, MS
119	1,10-di-*epi*-cubenol	0.1	0.2	-	-	1597	1615	RI, MS
120	135/146/159/71 M218	-	-	0.2	0.3	1602	-	RI, MS
121	muurola-4,10(14)-dien-1β-ol	0.2	0.2	-	-	1605	1620	RI, MS
122	gossonorol	-	-	0.1	0.1	1613	1626	RI, MS
123	**1-*epi*-cubenol**	0.2	**0.5**	-	-	1614	1623	RI, MS
124	**α-acorenol**	tr.	-	0.4	**0.5**	1620	1623	RI, MS
125	**τ-cadinol**	**1.4**	**4.1**	-	-	1625	1633	^1^H, RI, MS
126	τ-muurolol	0.3	0.4	-	-	1628	1633	RI, MS
127	**β-eudesmol**	**0.6**	0.2	**3.4**	**3.8**	1631	1641	RI, MS
128	**α-cadinol**	**1.3**	**3.8**	0.4	0.3	1638	1643	^1^H, RI, MS
129	5β,7βH,10α-eudesm-11-en- 1α-ol	-	-	0.3	-	1653	-	RI, MS
130	**6-methoxythymyl isobutyrate**	**0.9**	**0.7**	**3.8**	**4.9**	1657	1658	^1^H,^13^C, RI, MS
131	6-methoxy-8,9-didehydrothymyl isobutyrate	tr.	-	0.4	0.2	1665	1676	RI, MS
132	10-isobutyryloxy-8,9- didehydrothymol-methyl-ether	-	-	0.4	0.3	1666	1684	^1^H,^13^C, RI, MS
133	**α-bisabolol**	0.1	0.4	0.3	**1.3**	1668	1683	RI, MS
134	**145/162/71/115 M232**	-	-	0.3	**0.5**	1681	-	RI, MS
135	aromadendrene oxide	0.1	0.1	-	-	1702	1672	RI, MS
136	135/148/133/91 M236	-	-	0.1	0.1	1725	-	RI, MS
137	135/164/71/91 M234	-	-	0.2	0.1	1733	-	RI, MS
138	fenantrene (artifact)	0.1	0.3	-	-	1741	1744	RI, MS
139	diisobutylphtalate (artifact)	**0.9**	**2.5**	0.3	**0.6**	1817	1819	RI, MS
140	**hexahydrofarnesylacetone**	0.2	**0.8**	-	-	1820	1830	RI, MS
141	alantolactone	0.1	-	0.2	tr.	1854	1878	RI, MS
142	**9-isobutyryloxythymyl-** **isobutyrate**	0.2	**0.8**	**5.6**	**5.7**	1879	1891	^1^H,^13^C, RI, MS
143	**10-isobutyryloxy-8,9-** **didehydrothymyl-isobutyrate**	-	-	**3.0**	**3.1**	1882	1891	RI, MS
144	(5*E*,9*E*)-farnesylacetone	0.1	0.2	-	-	1889	1895	RI, MS
145	dibutylphtalate (artifact)	0.2	**1.8**	-	-	1906	1909	RI, MS
146	**7-isobutyryloxythymyl-** **isobutyrate**	-	-	**0.6**	**0.7**	1914	1924	RI, MS
147	**9-(2-methylbutyryloxy)thymyl-isobutyrate**	-	-	**1.0**	**1.4**	1964	1970	RI, MS
148	10-(2-methylbutyryloxy)-8,9- didehydrothymyl-isobutyrate	0.1	0.4	0.3	0.4	1967	1970	RI, MS
149	**10-isobutyryloxy-8,9-** **epoxythymyl-isobutyrate**	0.2	**0.6**	**29.2**	**18.1**	2002	2036	^1^H,^13^C,RI,MS
150	**71/177/150/135 M290**	-	-	**0.9**	**0.5**	2048	-	RI, MS
151	**10-(2-methylbutyryloxy)-8,9-** **epoxythymyl-isobutyrate**	-	-	**4.4**	**3.6**	2077	2056	RI, MS
152	10-isovaleroxy-8,9- epoxythymyl-isobutyrate	-	-	0.3	0.1	2097	2122	RI, MS
153	**fitol**	0.3	**1.7**	-	-	2098	-	RI, MS
154	57/177/71/85 M304	-	-	0.1	0.1	2149	-	RI, MS
155	tricosane	0.1	0.2	-	-	2286	2300	RI, MS
156	tetracosane	0.1	-	-	-	2386	2400	RI, MS
157	**pentacosane**	**0.6**	0.3	-	-	2489	2500	RI, MS
158	hexacosane	tr.	-	-	-	2589	2600	RI, MS
159	heptacosane	0.2	0.1	-	-	2685	2700	RI, MS
	**Sum of Identified**	**96.7**	**97.5**	**94.3**	**91.7**			
	**Yield of Essential Oil (%)**	**0.016**	**0.014**	**0.150**	**0.059**			

^a^ Essential oils isolated from aerial parts and roots of *Carpesium divaricatum* cultivated in the open field. ^b^ Essential oils isolated from aerial parts and roots of *Carpesium divaricatum* cultivated in a greenhouse. ^c^ Experimental retention index measured on non-polar column. ^d^ Literature retention index from non-polar column. ^e^ Identification based on retention index. ^f^ Identification based on mass spectrum. Tr.—<0.05%.

## Supplementary Materials

The following are available online: Figure S1: Mass spectra and retention indices (RI) together with chemical structures of thymol derivatives detected in *C. divaricatum* essential oils, Figure S2: Mass spectra and experimental retention indices (RI) of unidentified compounds from *C. divaricatum* essential oils, Figure S3: Results of NMR analyses of crude fractions (obtained by flash chromatography) from *C. divaricatum* essential oils.
