# Supplementary material for: Composition of Essential Oils from Roots and Aerial Parts of Carpesium divaricatum, a Traditional Herbal Medicine and Wild Edible Plant from South-East Asia, Grown in Poland
Source: Molecules. 2019 Dec 3;24(23):4418. doi: 10.3390/molecules24234418 (PMC6930655; doi:10.3390/molecules24234418)
Supplement: Supplementary file 1 [file molecules-24-04418-s001.pdf]

Composition of essential oils from roots and aerial parts of *Carpesium divaricatum*, a traditional herbal medicine and wild edible plant from South-East Asia, grown in Poland

Anna Wajs-Bonikowska [1], Janusz Malarz [2] and Anna Stojakowska [2,*]

[1] Institute of General Food Chemistry, Faculty of Biotechnology and Food Sciences, Łódź University of Technology, Stefanowskiego street 4/10, 90-924 Łódź, Poland; anna.wajs@p.lodz.pl

[2] Maj Institute of Pharmacology, Polish Academy of Sciences, Department of Phytochemistry, Smętna street 12, 31-343 Kraków, Poland; malarzj@if-pan.krakow.pl; stoja@if-pan.krakow.pl

* Correspondence: stoja@if-pan.krakow.pl; Tel.: +48 126623254

Supplementary material:

Figure S1. Mass spectra and retention indices (RI) together with chemical structures of thymol derivatives detected in *C. divaricatum* essential oils (the numbering of the compounds corresponds to that in Table 1) – pp. 2-9.

Figure S2. Mass spectra and experimental retention indices (RI) of unidentified compounds from *C. divaricatum* essential oils (the numbering of the compounds corresponds to that Table 1) - pp. 10-15.

Figure S3. Results of NMR analyses of crude fractions (obtained by flash chromatography) from *C. divaricatum* essential oils – pp. 16-21.

Figure S1. Mass spectra and retention indices (RI), together with chemical structures, of thymol derivatives detected in *C. divaricatum* essential oils (the numbering of the compounds corresponds to that in Table 1).

50. Thymol methyl ether (RI $_{exp.}$. 1211/RI $_{lit.}$ 1215)

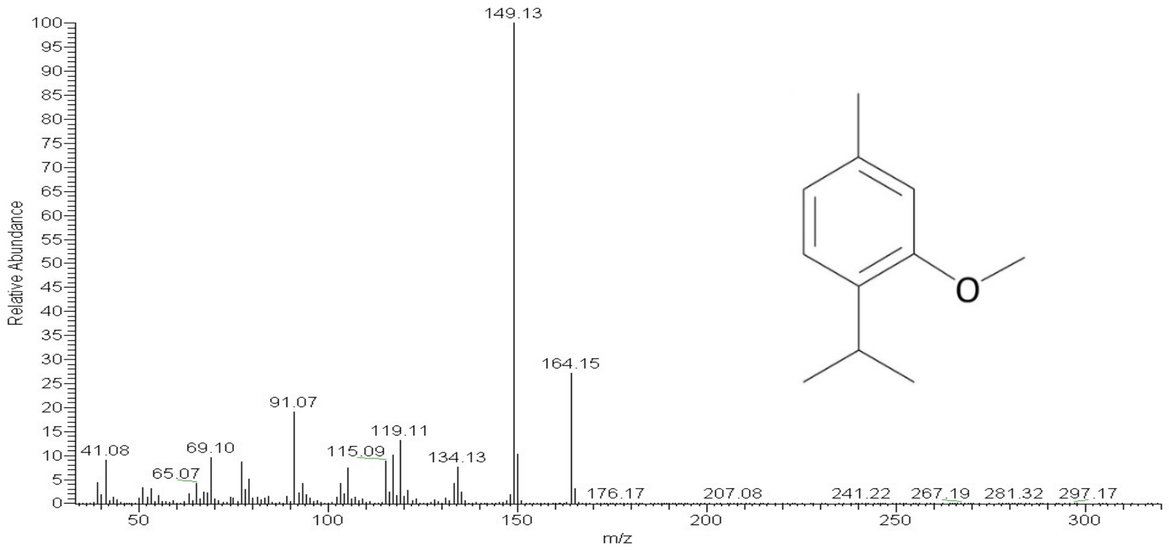

73. 6-Methoxythymol methyl ether (RI $_{exp.}$ 1394/RI $_{lit.}$ 1399)

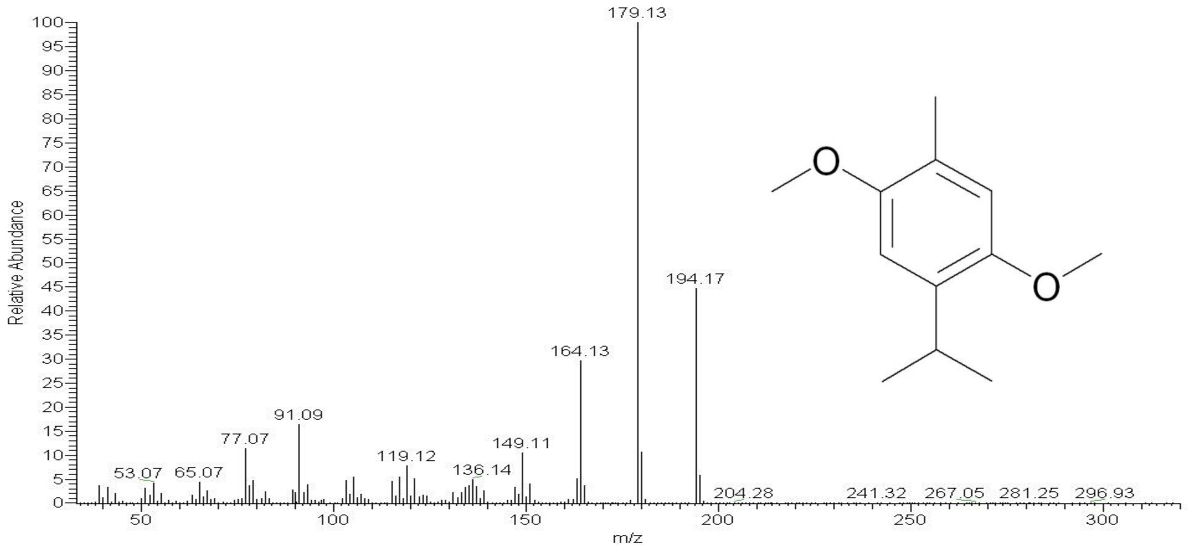

84. 8,9-Didehydrothymyl isobutryate (RI $_{exp.}$ 1461/RI $_{lit.}$ 1458)

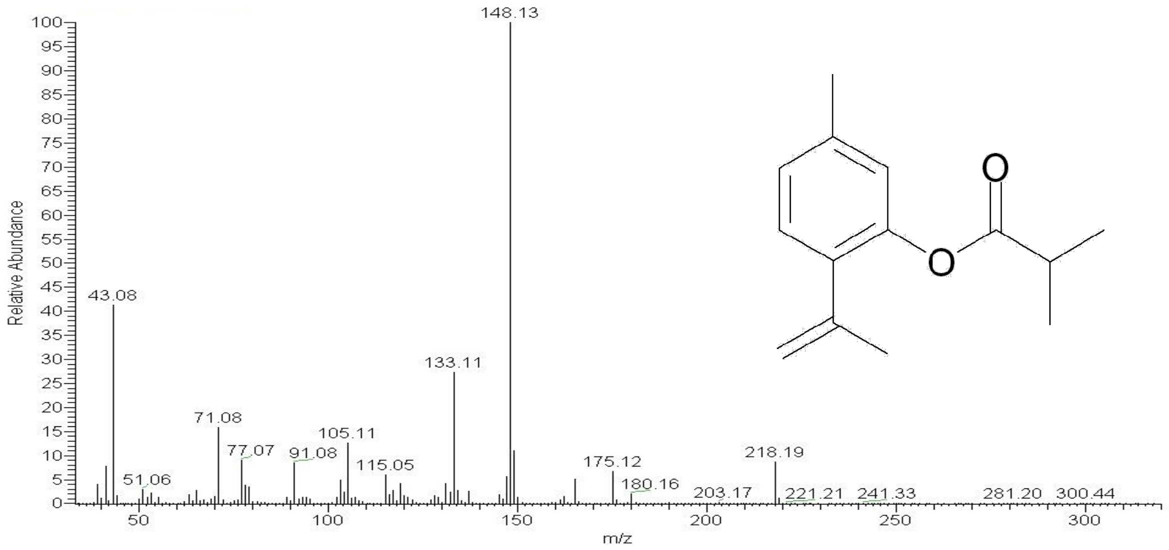

85. Thymyl isobutyrate (RI $_{exp.}$ 1467/RI $_{lit.}$ 1462)

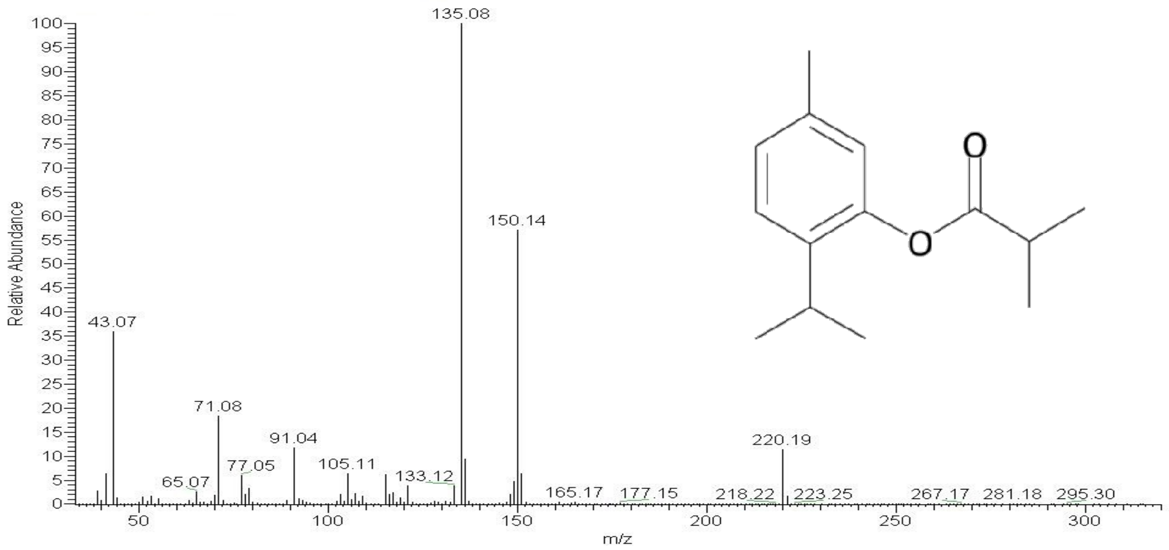

130. 6-Methoxythymyl isobutyrate (RI $_{exp.}$ 1657/RI $_{lit.}$ 1658)

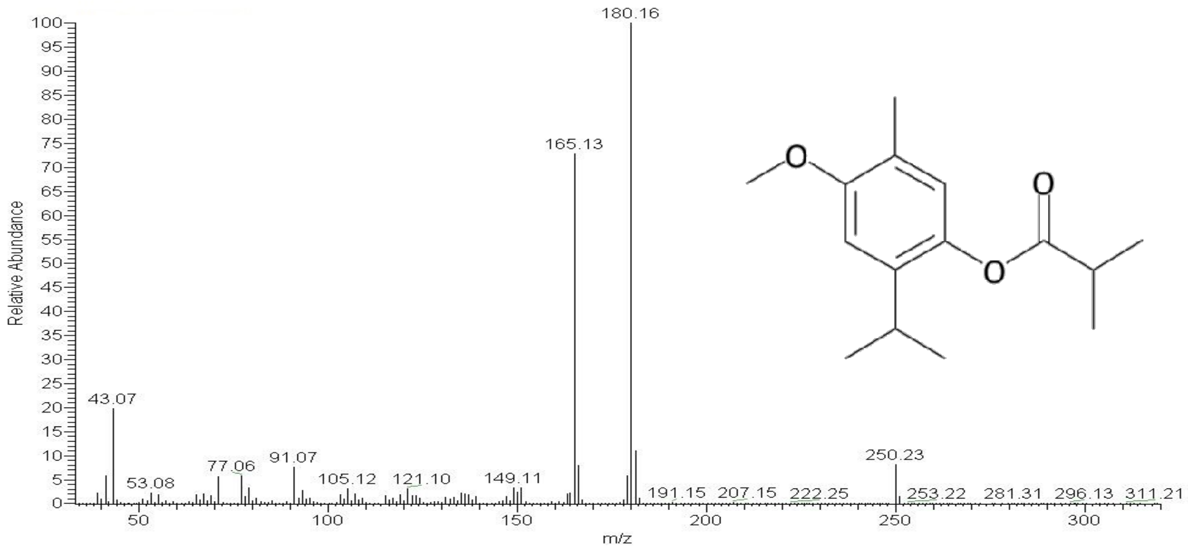

131. 6-Methoxy-8,9-didehydrotymyl isobutyrate (RI $_{exp.}$ 1665/RI $_{lit.}$ 1676)

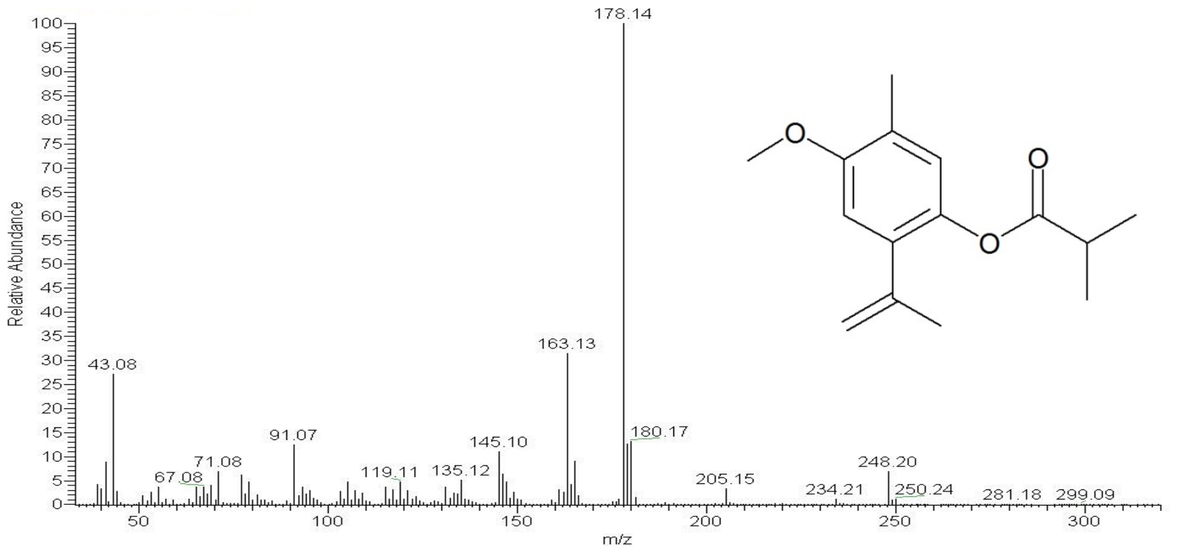

132. 10-Isobutyryloxy-8,9-didehydrothymol methyl ether (RI $_{exp.}$ 1666/RI $_{lit.}$ 1684)

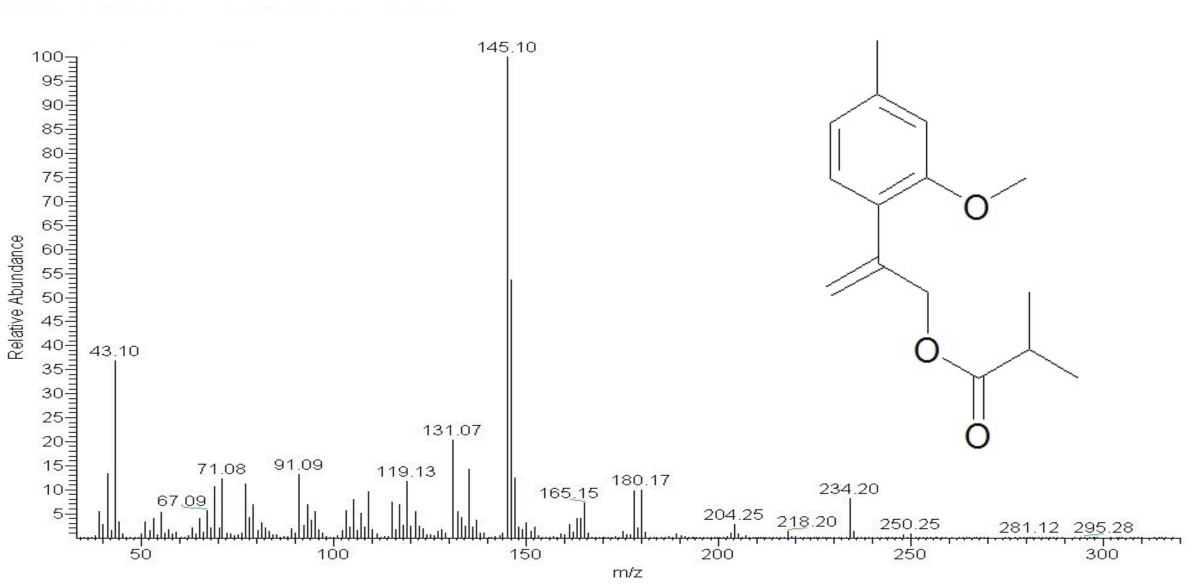

142. 9-Isobutyryloxytymyl isobutyrate (RI $_{exp.}$ 1879/RI $_{lit.}$ 1891)

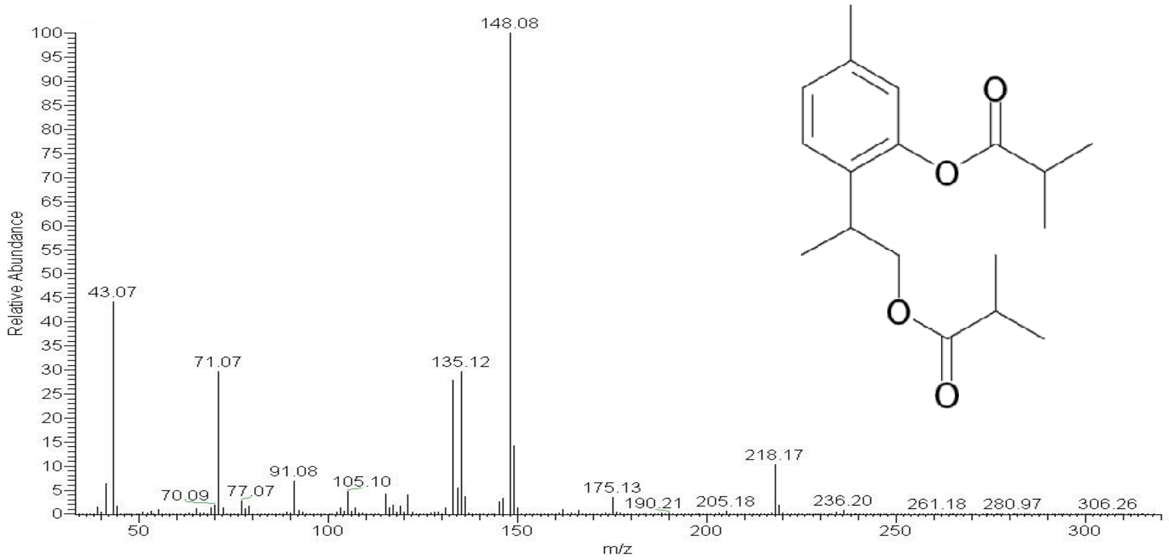

143. 10-Isobutyryloxy-8,9-didehydrothymyl isobutyrate (RI $_{exp.}$ 1882/RI $_{lit.}$ 1891)

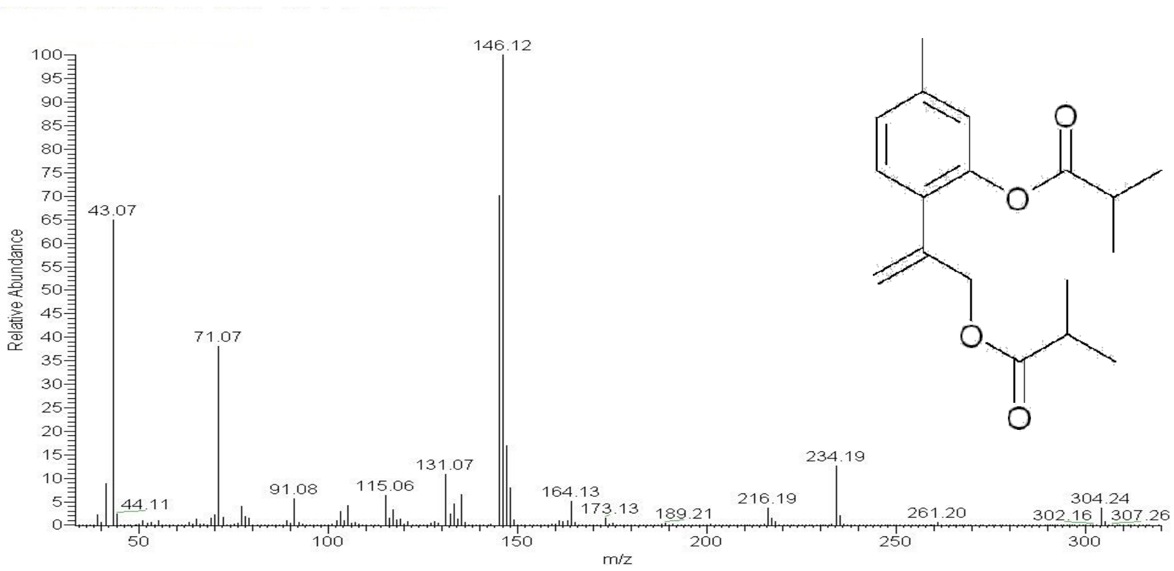

146. 7-Isobutyryloxythymyl isobutyrate (RI $_{exp.}$ 1914/RI $_{lit.}$ 1924)

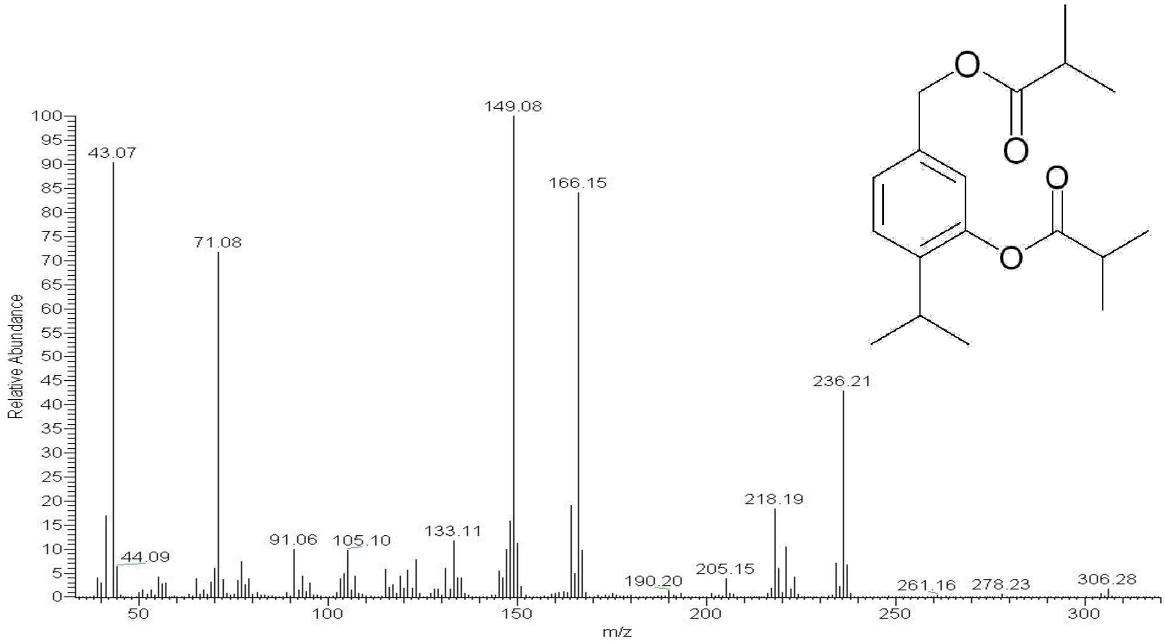

147. 9-(2-Methylbutyryloxy)thymyl isobutyrate (RI $_{exp.}$ 1964/RI $_{lit.}$ 1970)

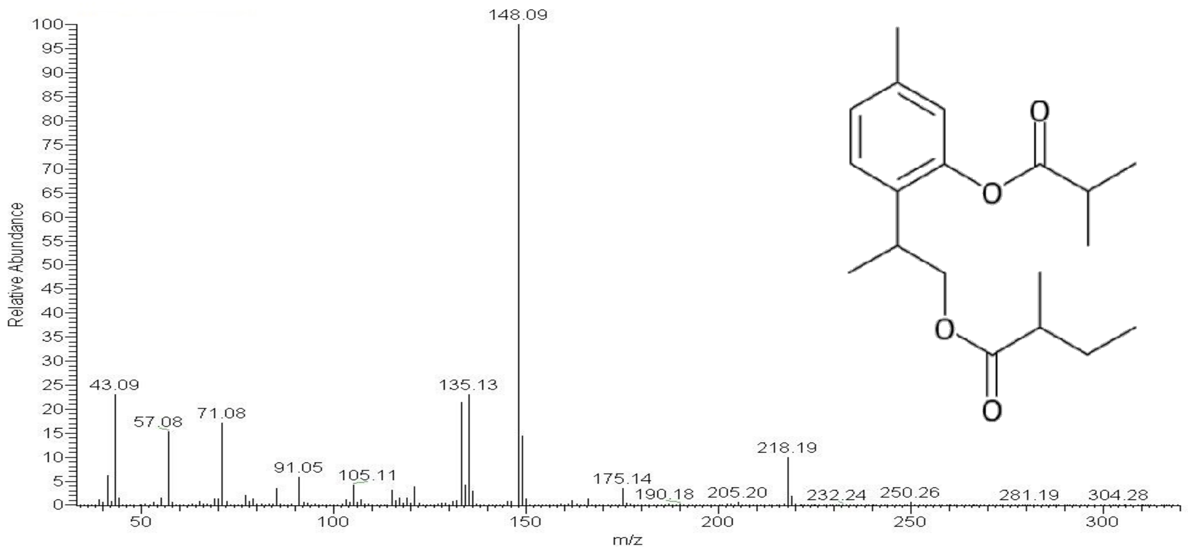

148. 10-(2-Methylbutyryloxy)-8,9-didehydrotymyl isobutyrate (RI $_{exp.}$ 1967/RI $_{lit.}$ 1970)

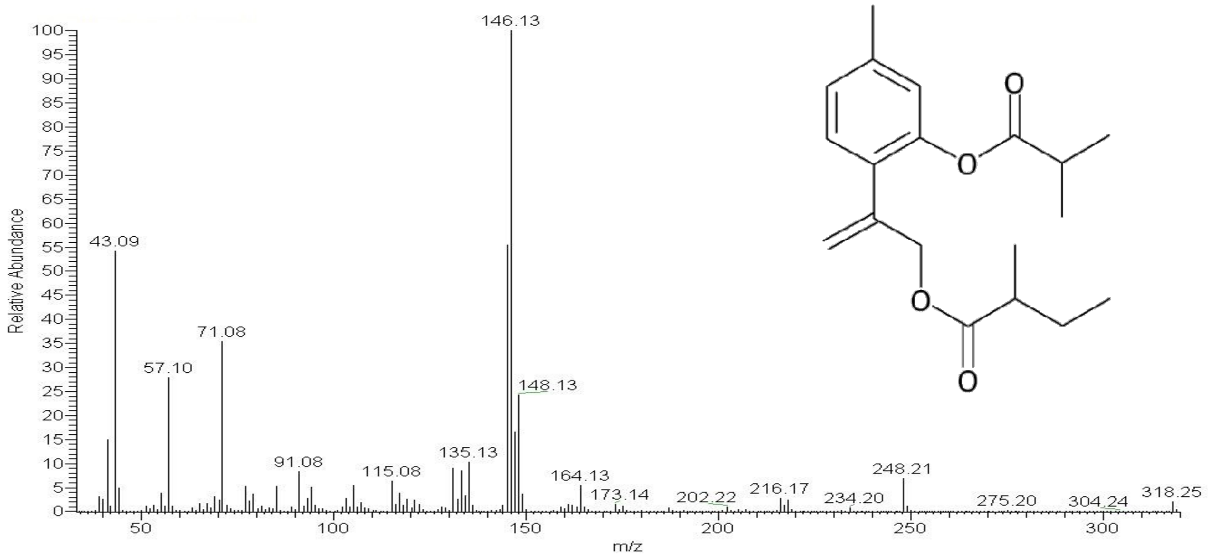

149. 10-Isobutyryloxy-8,9-epoxythymyl isobutyrate (RI $_{exp..}$ 2002/RI $_{lit.}$ 2036 [HP-5 column])

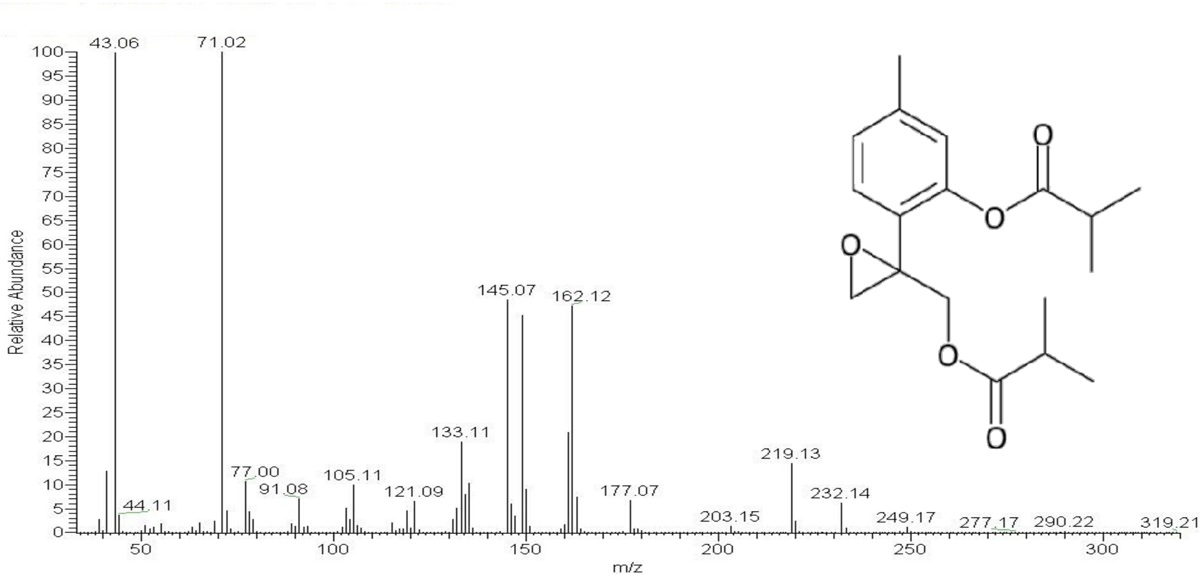

151. 10-(2-Methylbutyryloxy)-8,9-epoxythymyl isobutyrate (RI $_{exp.}$ 2077/RI $_{lit.}$ 2056 [BP-1 column])

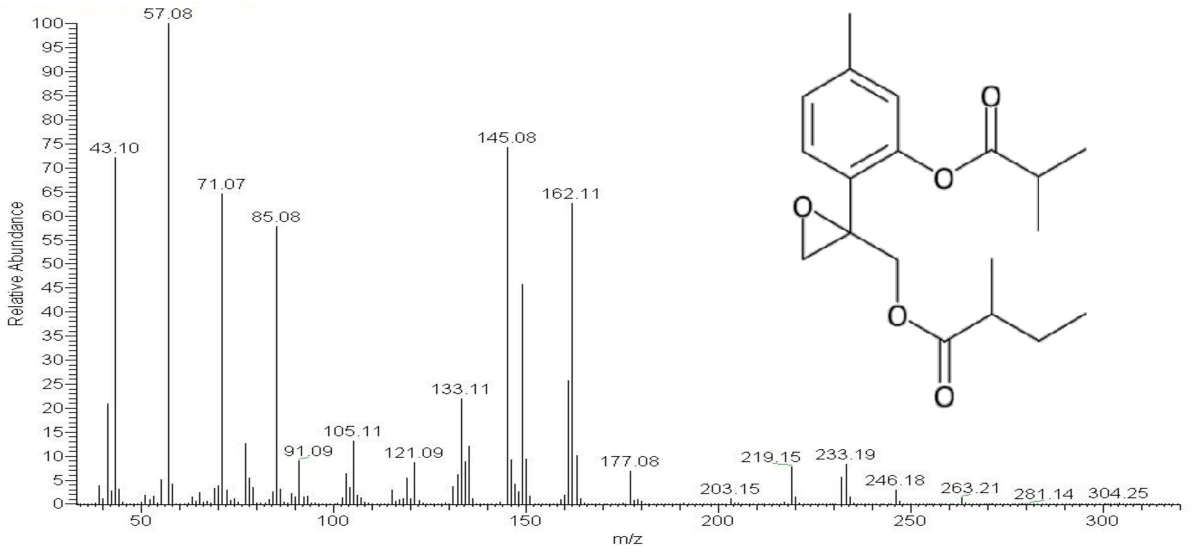

152. 10-Isovaleroxy-8,9-epoxythymyl isobutyrate (RI $_{exp.}$ 2097/RI $_{lit.}$ 2122 [HP-5 column])

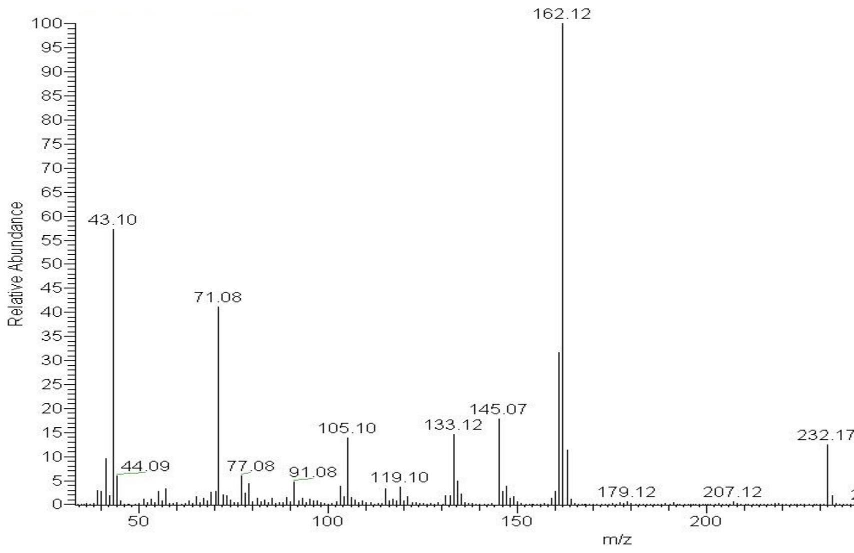

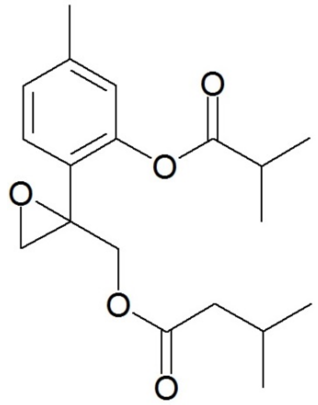

Figure S2. Mass spectra and experimental retention indices (RI) of unidentified compounds from *C. divaricatum* essential oils (the numbering of the compounds corresponds to that in Table 1).

Compound 25: RI $_{exp.}$ 1091

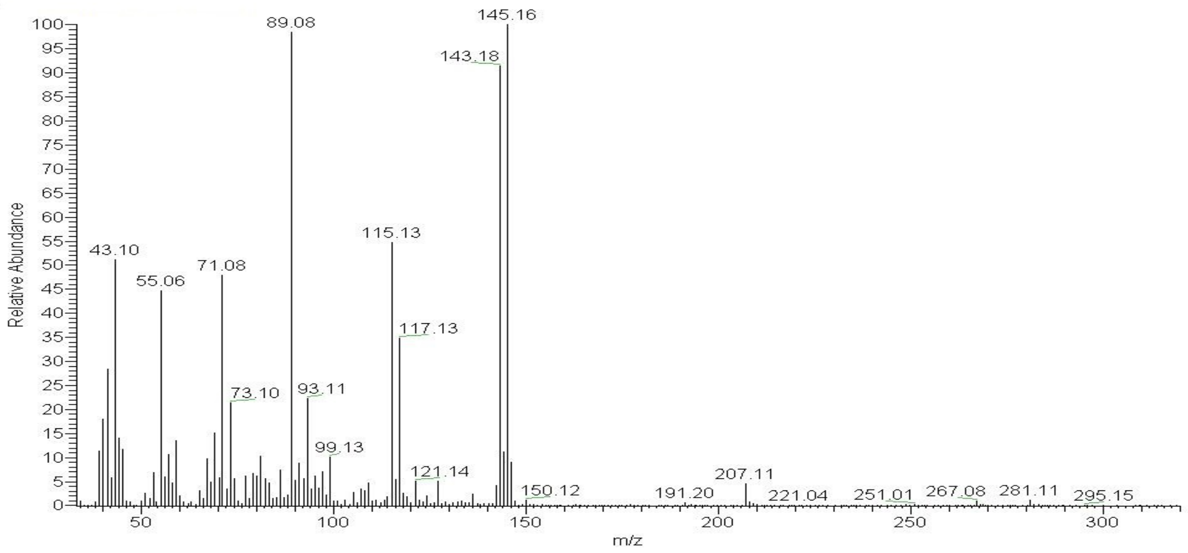

Compound 72: RI $_{exp.}$ 1391

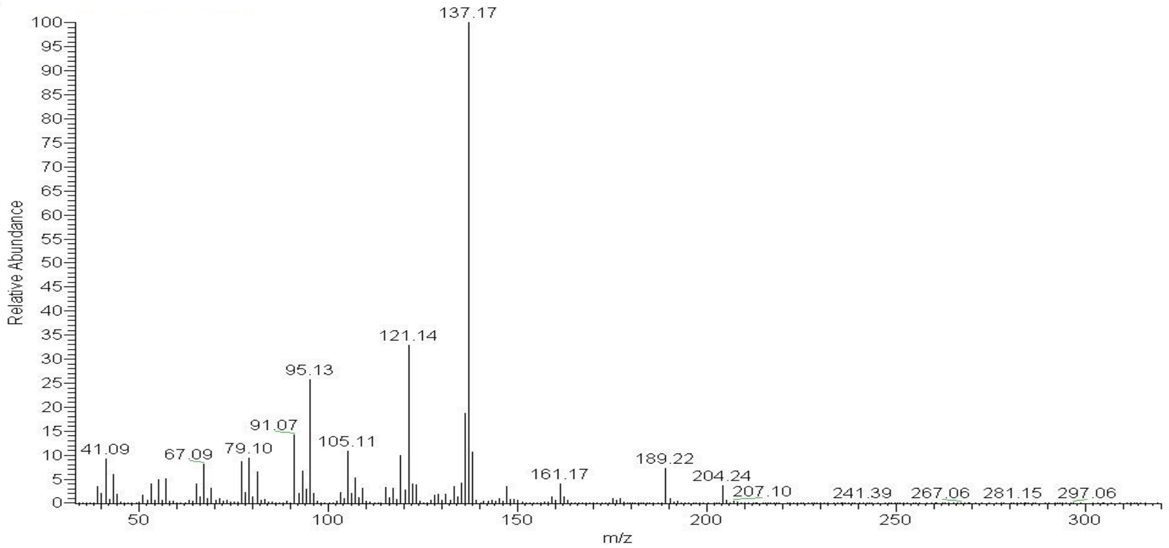

Compound 89: RI $_{exp.}$1484

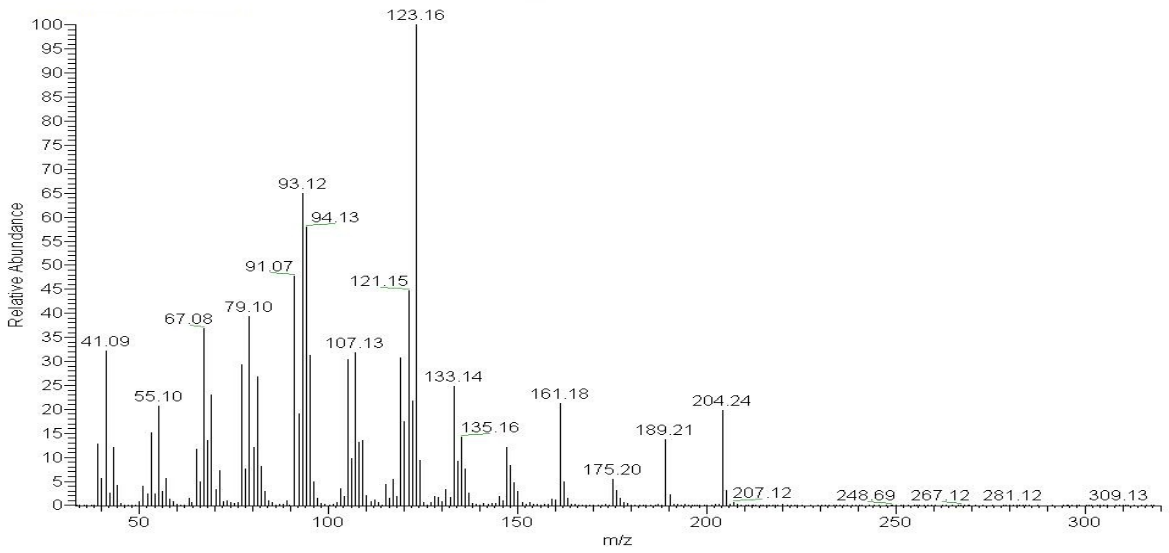

Compound 106: RI $_{exp.}$1531

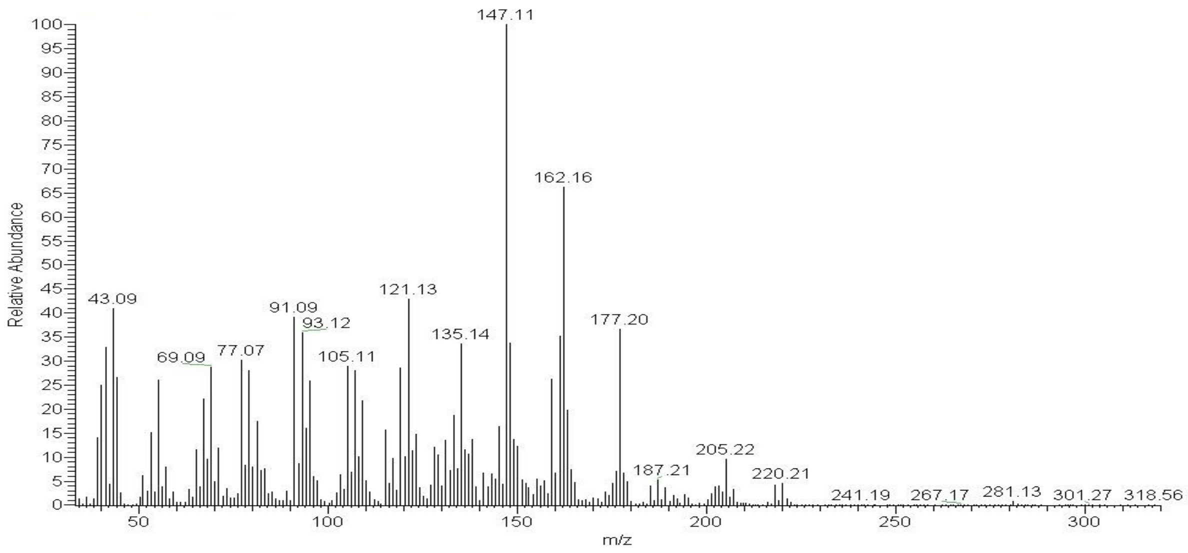

Compound 107: RI $_{exp.}$1534

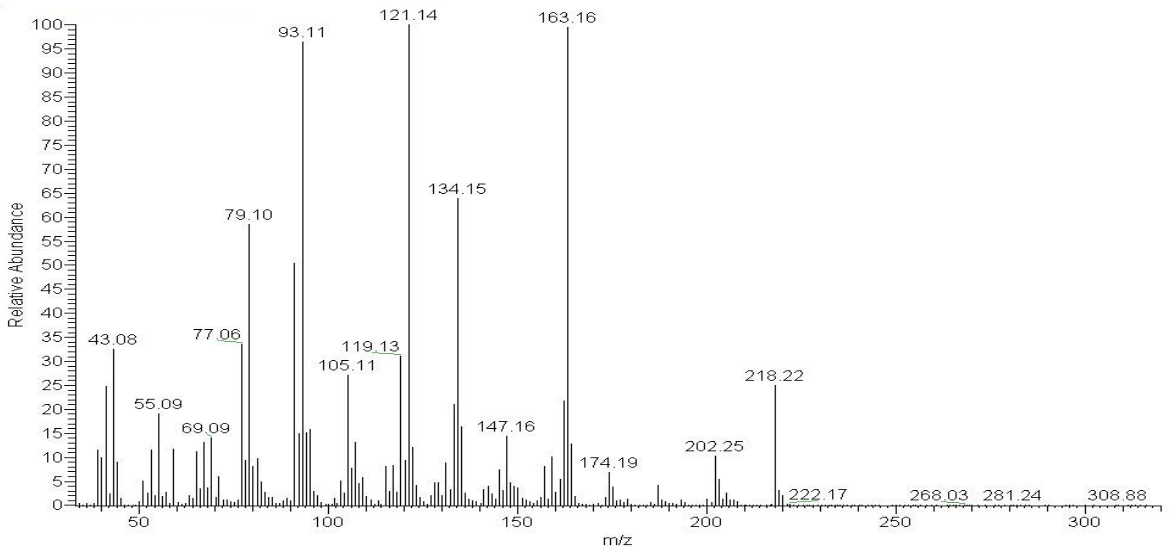

Compound 120: RI $_{exp.}$1602

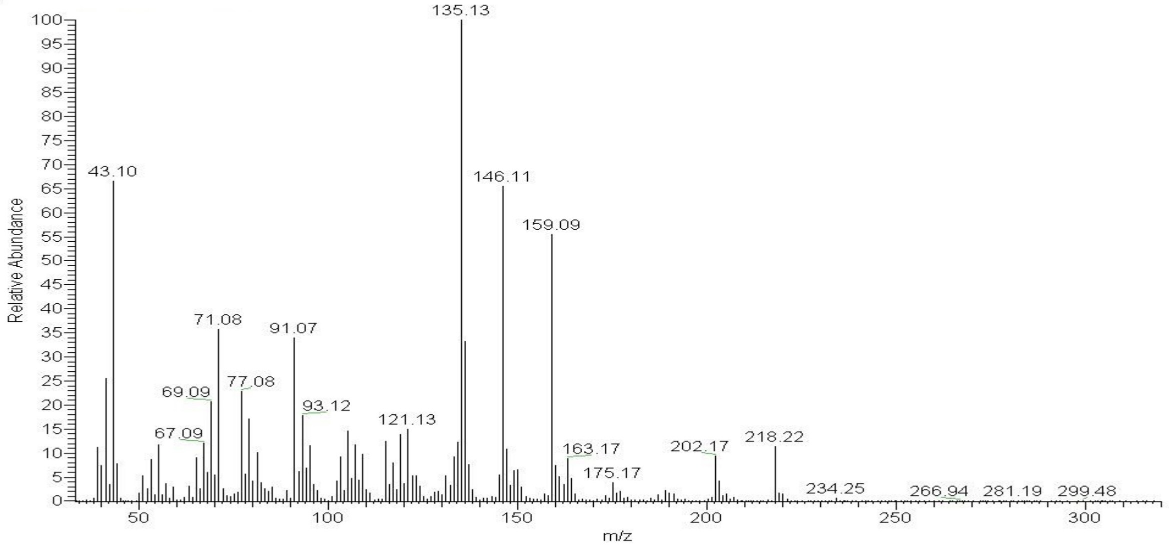

Compound 134: RI $_{exp.}$ 1681

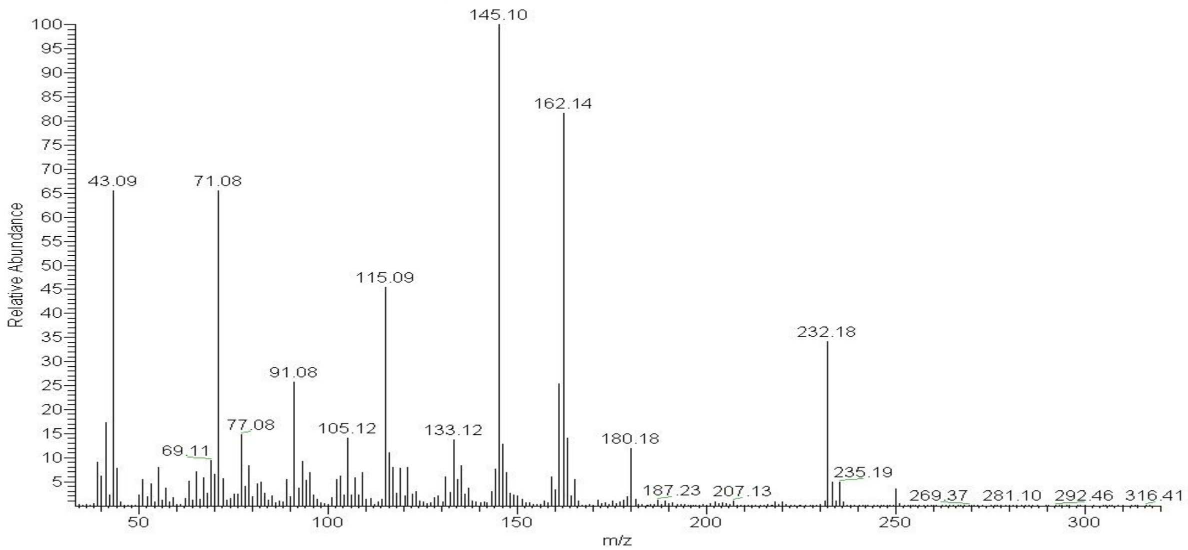

Compound 136: RI $_{exp.}$ 1725

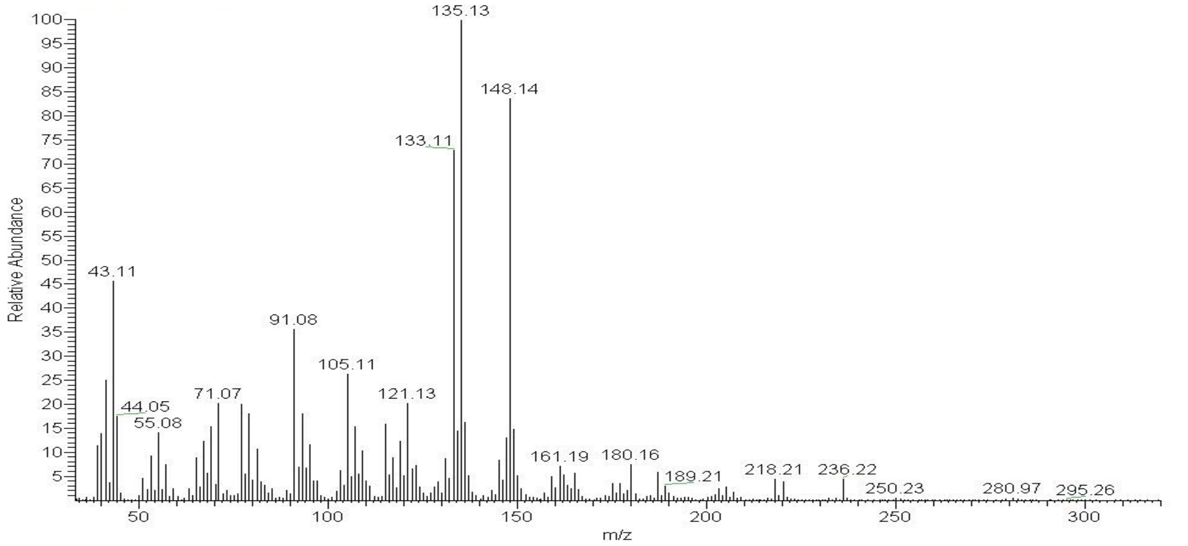

Compound 137. RI $_{exp.}$ 1733

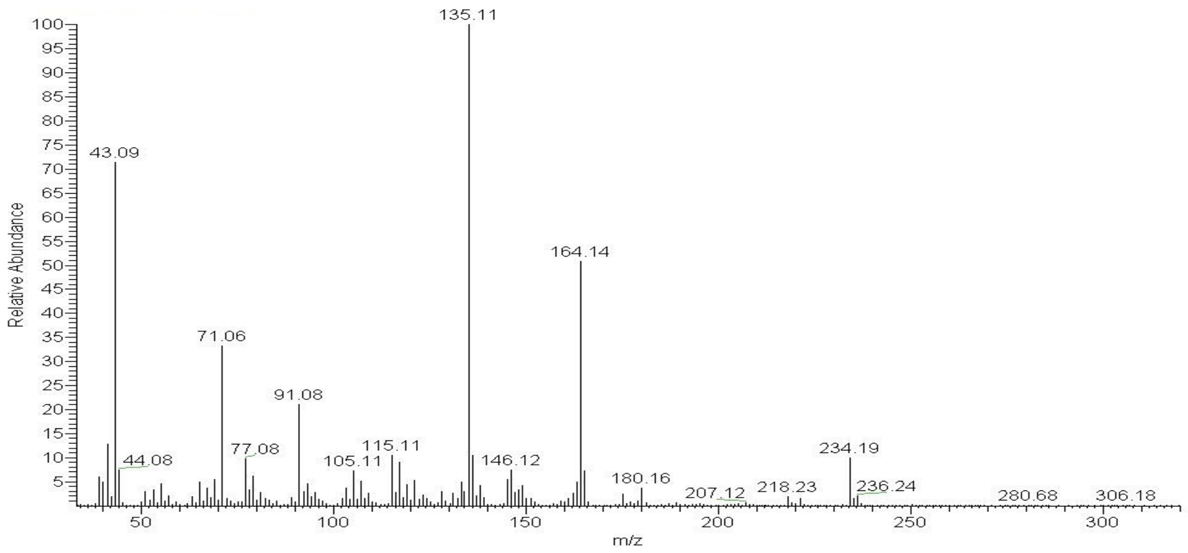

Compound 150: RI $_{exp.}$ 2048

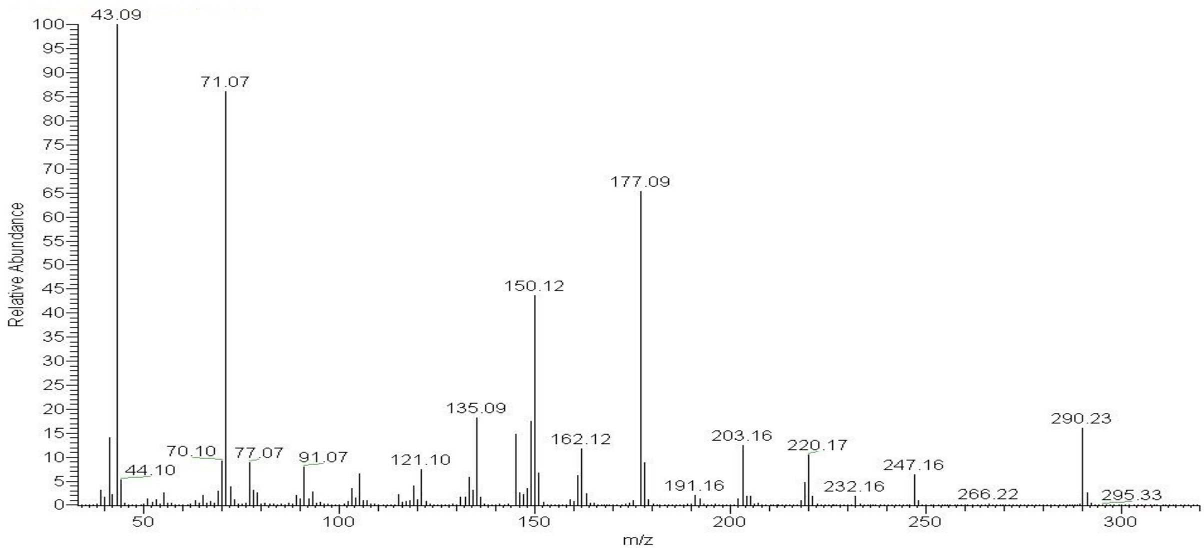

Compound 154: RI $_{exp.}$ 2149

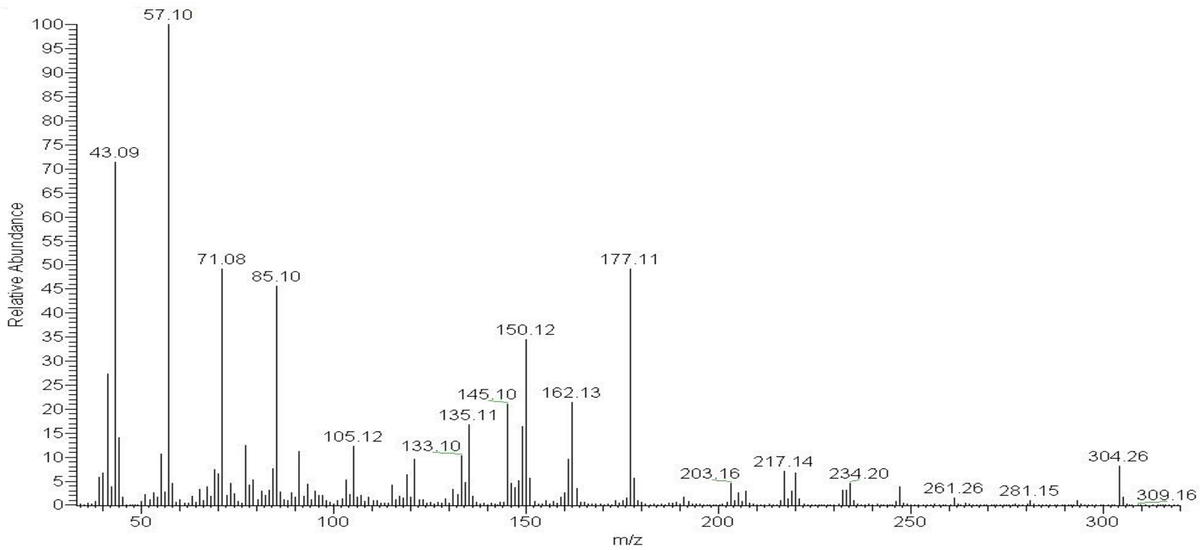

Figure S3. Results of NMR analyses of crude fractions (obtained by flash chromatography) from *C. divaricatum* essential oils (chemical shifts given in the literature are printed in blue).

Fraction 1a: neryl isobutyrate (25.5% of the fraction)

- **^{1}H-NMR**

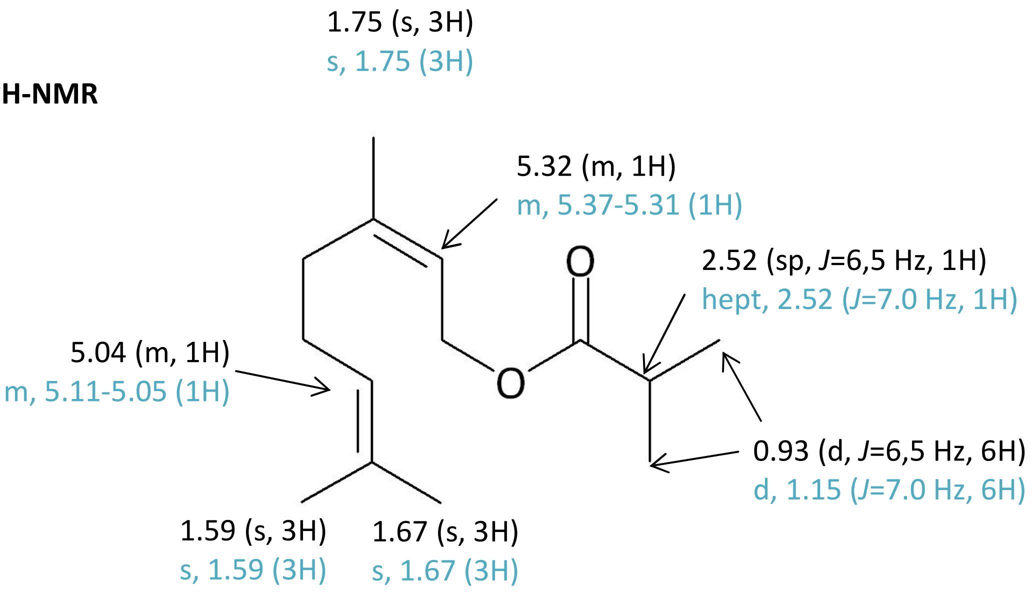

Fraction 13b: (*E*)-β-caryophyllene oxide (51.5% of the fraction)

- **^{1}H-NMR**

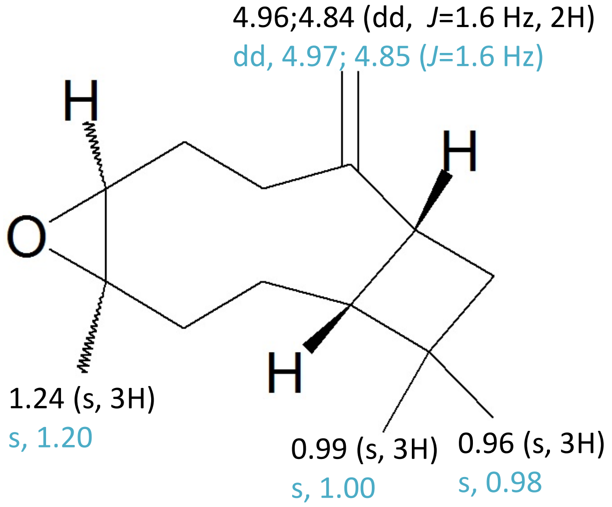

Fraction 13a: (*E*)-nerolidol (24.6% of the fraction)

- **^{1}H-NMR**

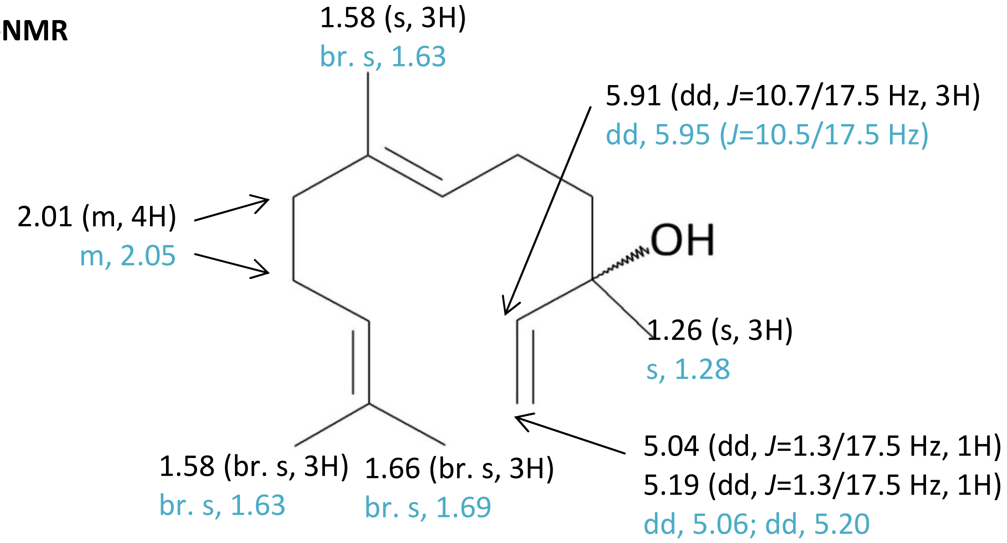

Fraction 18b: τ-cadinol (20,7% of the fraction)

- **^{1}H-NMR**

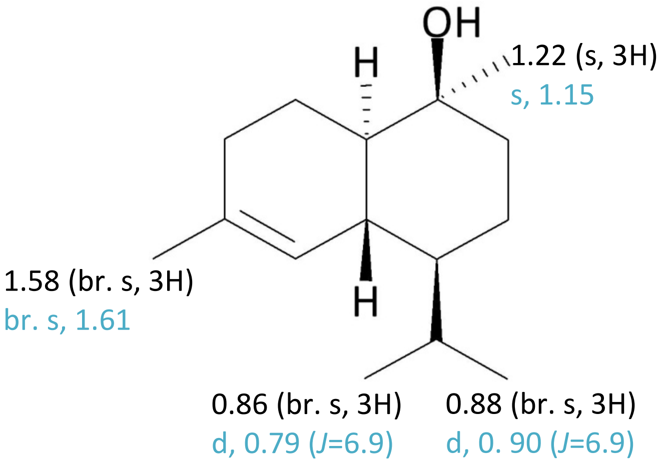

Fraction 17a: nerol (25,2% of the fraction)

- **^{1}H-NMR**

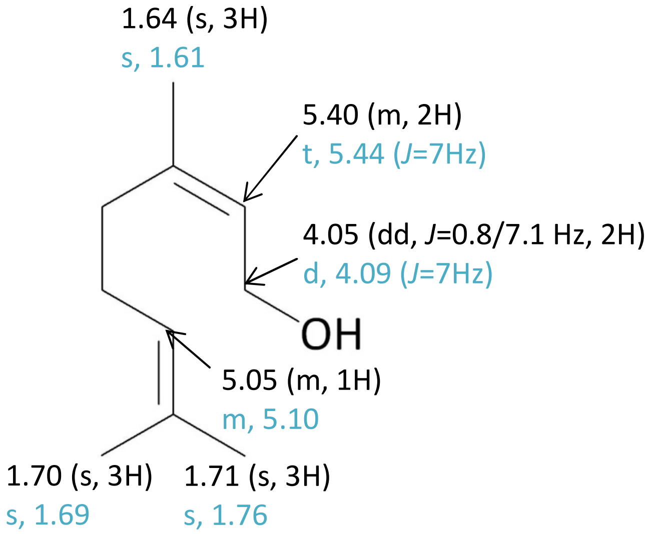

Fraction 18a: α-cadinol (22.8% of the fraction)

- **^{1}H-NMR**

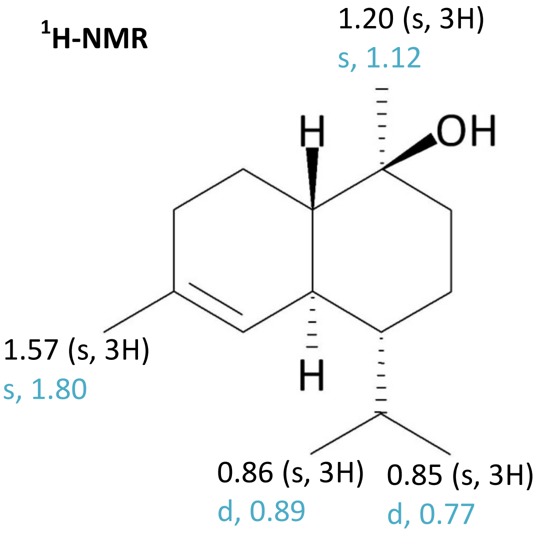

Fraction 3b: thymol methyl ether (33.0% of the fraction)

- **^{1}H-NMR**

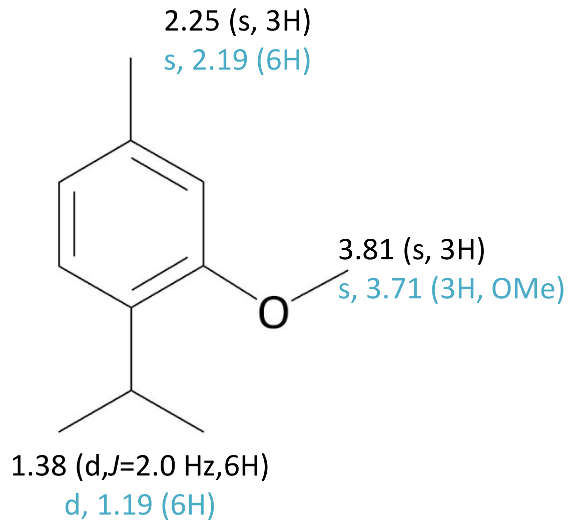

Fraction 7b: thymyl isobutyrate (57.2% of the fraction)

- **^{1}H-NMR**

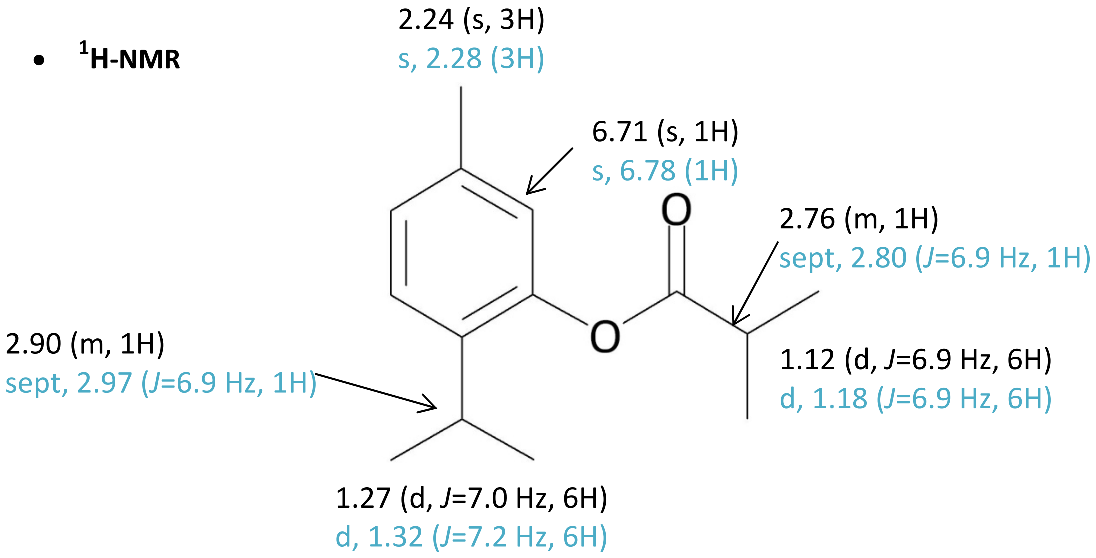

Fraction 14b: 10-isobutyryloxy-8,9-didehydrothymyl isobutyrate (45,5%)

^{13}C-NMR

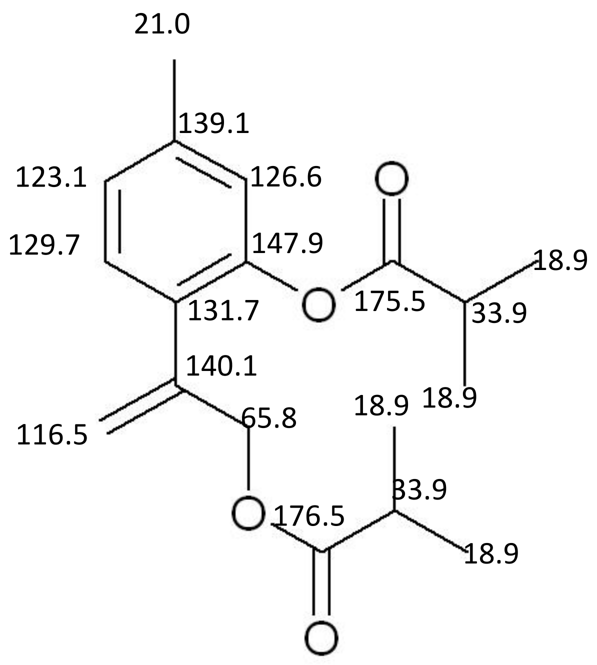

Fraction 15 b: 9-isobutyryloxythymyl isobutyrate (54.6%)

- **^{13}C-NMR**

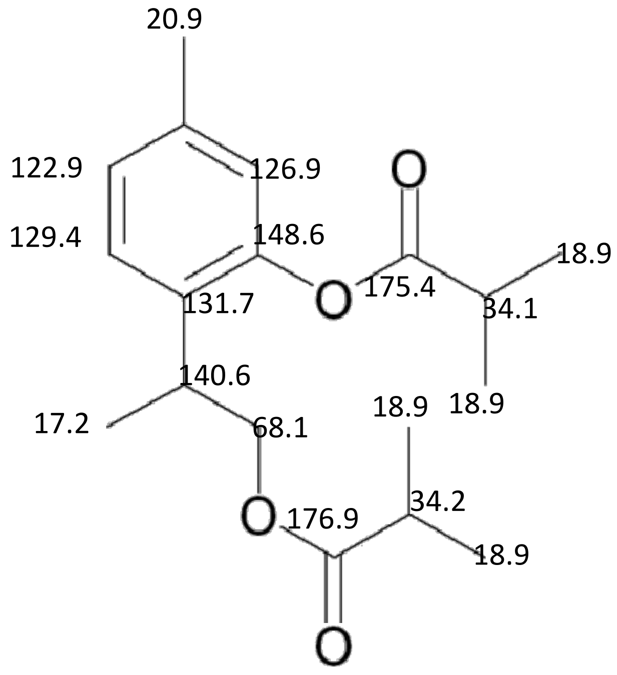

Fraction 17 b: 10-isobutyryloxy-8,9-epoxytymyl isobutyrate (54.6%)

^{1}H-NMR: δ 7.35 (d, J=7.8Hz, 1H), 7.03 (d, J=7.8 Hz, 1H), 6.86 (s, 1H), 4.56 (d, J=12.2 Hz, 1H), 4.18 (d, J=12.2 Hz, 1Hz), 3.02 (d, J=5.4Hz, 1H), 2.85 (hept.,1H), 2.78 (d, J=5.4Hz, 1H), 2.50 (hept.1H), 2.33 (s, 3H), 1.32 (d, J=7.0 Hz, 6H), 1.10 (d, J=7.O Hz, 3H), 1.07 (s, J=7.0 Hz, 3H)

^{13}C-NMR: δ 175.4 (C1′), 174.3 (C1″), 147.6 (C3), 138.9 (C1), 127.9 (C5), 125.7 (C6), 125.0 (C4), 121.9 (C2), 63.9 (C10), 55.9 (C8), 49.7 (C9), 33.2 (C2′), 32.8 (C2″), 19.9 (C7), 17.9 (C3′,C4′,C3″,C4″)

Literature:

Joulain, D.; König, W. The Atlas of Spectral Data of Sesquiterpene Hydrocarbons. 1998. Hamburg, Germany E.B. Verlag.

Anthonsen, T.; Kjøsen B. New thymol derivatives from *Inula salicina* L. Acta Chem. Scand. 1971, 25, 390-392.

Mathela, C.S.; Tiwari, A.; Padalia, R.C.; Chanotija, C.S. Chemical composition of *Inula cuspidata* C.B. Clarke. Indian J. Chem. B 2008, 47, 1249-1253.

Bohlmann, F.; Niedballa, U.; Schulz, J. Über einige Thymolderivate aus *Gaillardia*- und *Helenium*-Arten. Chem. Ber. 1969, 102, 864-871.

Bohlmann, F.; Mahanta, P.K.; Suwita, A.; Natu, A.A.; Zdero, C.; Dorner, W.; Ehlers, D.; Grenz, M. Neue Sesquiterpenlactone und andere Inhaltsstoffe aus Vertretern der Eupatorium-gruppe. Phytochemistry 1977, 16, 1973-1981.

Weremczuk-Jeżyna, I.; Wysokińska, H.; Kalemba, D. Constituents of the essential oil from hairy roots and plant roots of *Arnica montana* L. J. Essent. Oil Res. 2011, 23(1), 91-97.

Zee, O.P.; Kim, D.K.; Lee, K.R. Thymol derivatives from *Carpesium divaricatum*. Arch. Pharm. Res. 1998, 21, 618-620.
